# Functional Annotation and Comparative Analysis of a Zygopteran Transcriptome

**DOI:** 10.1534/g3.113.005637

**Published:** 2013-04-01

**Authors:** Alexander G. Shanku, Mark A. McPeek, Andrew D. Kern

**Affiliations:** * Rutgers, The State University of New Jersey, Department of Genetics, Piscataway, New Jersey 08854-8082; † Department of Biological Science, Dartmouth College, New Hampshire 03755

## Abstract

In this paper we present a *de novo* assembly of the transcriptome of the damselfly (*Enallagma hageni*) through the use of 454 pyrosequencing. *E. hageni* is a member of the suborder Zygoptera, in the order Odonata, and Odonata organisms form the basal lineage of the winged insects (Pterygota). To date, sequence data used in phylogenetic analysis of *Enallagma* species have been derived from either mitochondrial DNA or ribosomal nuclear DNA. This *Enallagma* transcriptome contained 31,661 contigs that were assembled and translated into 14,813 individual open reading frames. Using these data, we constructed an extensive dataset of 634 orthologous nuclear protein-encoding genes across 11 species of Arthropoda and used Bayesian techniques to elucidate the position of *Enallagma* in the arthropod phylogenetic tree. Additionally, we demonstrated that the *Enallagma* transcriptome contains 169 genes that are evolving at rates that differ relative to those of the rest of the transcriptome (29 accelerated and 140 decreased), and, through multiple Gene Ontology searches and clustering methods, we present the first functional annotation of any palaeopteran’s transcriptome in the literature.

*Enallagma* damselflies are aquatic invertebrates belonging to the order Odonata. Included in this group are dragonflies (suborder Anisoptera) and other damselflies (suborder Zygoptera), which together represent one of the most ancient branches of the winged insects (Pterygota) and furthermore represent a basal group within the division Palaeoptera (Simon *et al.* 2009). The damselfly has a rich history as an organism used in evolutionary and ecological studies, spanning research in speciation (Bourret *et al.* 2011; Turgeon *et al.* 2005), species distribution (Bourret *et al.* 2011), selection (Outomuro *et al.* 2011; Abbott *et al.* 2008), population diversity (Iserbyt *et al.* 2010), and predator-prey interactions (Mittelbach *et al.* 2007; Slos *et al.* 2009; Strobbe *et al.* 2010).

Despite the fact that this organism is an ideal candidate for many types of biological studies, there has been relatively little examination of the genetic makeup of damselflies on a large scale (Bellin *et al.* 2009; Surget-Groba and Montoya-Burgos 2010; Nawy 2011). For example, most of the sequence data used to determine phylogenetic relationships among *Enallagma* species, as well as to infer *Enallagma* phylogenetic relationships within Odonata, has been in the form of mtDNA (Turgeon and McPeek 2002; Saux *et al.* 2003) or ribosomal nuclear DNA (Dumont and Vierstraete 2010). Therefore, in this study, we attempted to investigate the nuclear, protein-encoding gene profile of the damselfly *Enallagma hageni* by using next-generation sequencing technology and, by doing so, (1) give further resolution and support to this organism’s phylogenetic position within Arthropoda, (2) determine the evolutionary rates of the protein-encoding genes in the *Enallagma* transcriptome, and (3) give functional annotation to the proteins expressed in our dataset.

## Materials and Methods

### Insect capture and RNA preparation

Individuals across the entire life cycle were included in the sample from which RNA was extracted. Some *Enallagma* larvae are difficult to identify as to species, with *E. hageni* being one of these. *E. hageni* larvae are largely indistinguishable from those of four other species that are all derived from a very recent radiation (Turgeon *et al.* 2005). To ensure that we were unambiguously collecting *E. hageni* larvae, we collected larvae from Martin’s Pond, Green Bay, VT, a lake where we have found only *E. hageni* and none of the other species as adults in previous years (M. A. McPeek, personal communication). Embryos were obtained by allowing females to oviposit in the laboratory and then allowing 2 weeks for development prior to RNA extraction. Aquatic larvae from across the entire range of the larval period and adults were collected and immediately placed in RNAlater (Ambion Inc.) until RNA isolation. Total RNA was isolated from the pooled material of roughly 50 embryos, 150 larvae, and 25 adults by first flash freezing the insects in liquid nitrogen and then processing the frozen material using RNeasy protocols (Qiagen). From our isolations, we collected roughly 100 mg of total RNA.

### Transcriptome sequencing and assembly

mRNA isolation, library construction, and 454 sequencing were contracted out to Beckman Coulter Genomics, using 1 mg of total RNA as starting material. All sequencing was of un-normalized cDNA libraries, using standard 454 protocols with the 454GS instrument. This produced 976,767 reads (see *Results* for details of the sequencing output).

To perform *de novo* transcriptome assembly with our reads, we used the Newbler assembler (version 2.3) using parameter settings specifically for mRNA assembly (see Supporting Information, Table S5).

### Protein translation

To compile a dataset of proteins which would form the basis of our analysis, assembled contigs were translated using Virtual Ribosome (Wernersson 2006). Each of 6 open reading frames (ORFs) was translated (where –readingframe = all), and the longest resulting translated read was kept, provided it was initiated with a start codon (where –orf = any). To account for contigs that might have had their upstream start codon truncated during assembly, we again translated more than 6 ORFs, all contigs that did not posses a start codon but terminated with a stop codon (where –orf = none). Of these two sets of putative proteins, the longest read that possessed both a start and a stop codon was determined to be the translated protein for a given contig, unless it was a fragment not initiated by a start codon but terminated with a stop codon, was greater in length. Contigs composed of fewer than 10 nucleotides were excluded from translation and removed from further analysis.

### Arthropod proteins

Comparative analysis of phylogenetic relationships necessitates alignment of homologous sequences among individuals being compared. To compile the data for such an analysis, we began by conducting a search aimed at identifying orthology across expressed proteins in a group of selected arthropods. To build this set of putative orthologous proteins, we obtained transcriptome data from ten arthropod species housed in public databases (Table 1 and Figure S1).

**Table 1:** Arthropod Species Used in Phylogenetic Analysis

Binomial Name	Common Name	Class/Order	Public Database
*Acythosiphon pisum*	Pea aphid	Insecta/Hemiptera	NCBI
*Anopheles gambiae*	Mosquito	Insecta/Diptera	Vectorbase
*Apis mellifera*	Honey bee	Insecta/Hymenoptera	NCBI
*Bombyx mori*	Silkworm	Insecta/Lepidoptera	Silkworm Genome Database
*Camponotus floridanus*	Carpenter ant	Insecta/Hymenoptera	Hymenoptera Genome Database
*Daphnia Pulex*	Water flea	Branchiopoda/Cladocera	wFleaBase (Daphnia Genome Project)
*Drosophila melanogaster*	Fruit fly	Insecta/Diptera	Flybase
*Ixodes scapularis*	Deer tick	Arachnida/lxodida	Vectorbase
*Pediculus humanus*	Body louse	Insecta/Phthiraptera	Vectorbase
*Tribolium castaneum*	Red flour beetle	Insecta/Coleoptera	NCBI

These 10 species’ transcriptomes were obtained from publicly accessible databases. Included in this dataset are 1 arachnid, 1 branchiopod, and 8 insect classes. All data were downloaded from their respective databases in January 2011.

### Ortholog Detection

To construct a working set of orthologous proteins, we used the method of one-to-one reciprocal best BLAST hits (Moreno-Hagelsieb and Latimer 2008; Gabaldón 2008), rather than attempt to use ortholog clustering methods (*e.g.*, OrthoMCL) (Li *et al.* 2003). We used BLAST to search for protein-encoding genes between each species’ transcriptome and those in *D. melanogaster*, and conversely, the *D. melanogaster* transcriptome was also searched using BLAST for all protein-encoding genes present in each of the species’ transcriptomes in the dataset. The best hit was determined using the “-K 1” and “-b 1” BLAST parameters, which limit output, in this case the “-m 8” tabulated output format, to the best scoring hit of each BLAST query. Following this methodology and using mpiBLAST, an open-source, parallelized version of BLAST (Darling and Carey 2003), we constructed a set of reciprocal-best, one-to-one orthologs. To expedite computational processing time, each species’ database file was partitioned into 94 fragments (where *n*frags = 94), and the parameter setting “–output-search-stats–use-parallel-write–use-virtual-frags–removedb” was used for each job. Using customized scripts, individual orthologs that were present across all 11 arthropod species were grouped together into individual .fasta files. Following this search and grouping method, the protein sequences within each file were aligned using ClustalW2 software using the flags “-OUTPUT=FASTA” and “-OUTORDER=INPUT,” the latter being necessary to later allow for concatenation of all aligned orthologs when conducting phylogenetic analysis (Larkin *et al.* 2007).

### Phylogenetics

Each orthologous gene alignment was concatenated into a “super-gene” (Gadagkar *et al.* 2005), that is, we took individual .fasta files and joined them into one singular, interleaved .nexus file by using a customized Ruby script. If an amino acid position in the concatenated alignment contained a gap at a position in any of the species, or in multiple species, that position was removed prior to analysis by using Gblocks 0.91b (Talavera and Castresana 2007), as we did not use a model of sequence evolution that allowed for insertions or deletions.

### Model selection

To determine the optimal model of protein evolution for phylogenetic analysis of our dataset, ProtTest version 2.4 software was used for model selection (Darriba *et al.* 2011; Abascal *et al.* 2005). All amino acid evolutionary rate models available in ProtTest were examined, as were the parameters “+I,” “+G,” and “+F”. (Dayhoff *et al.* 1978), JTT (Jones *et al.* 1992), WAG (Whelan and Goldman 2001), mtREV (Adachi and Hasegawa 1996), MtMam (Cao *et al.* 1994), VT (Müller and Vingron 2000), CpREV (Adachi *et al.* 2000), RtREV (Dimmic *et al.* 2002), MtArt (Abascal *et al.* 2007), HIVb/HIVw (Nickle *et al.* 2007), LG (Le and Gascuel 2008), and Blosum62 (Henikoff 1992).

Ideally, we would optimize tree topology, branch lengths, and parameters of the model for each model investigated. This was inefficient in our case, as the dataset is too large to realistically attempt topology optimization for each model and each additional model parameter associated with that model. Instead, we allowed a neighbor-joining tree to be constructed with our data, and fix the topology and from that topology, optimize branch lengths and select model parameters (Posada and Crandall 2001).

### Bayesian phylogenetic inference

Once the optimum model was selected, we searched topology space of the 11 arthropod species in our dataset with a Bayesian Markov chain Monte Carlo (MCMC) approach using MrBayes version 3.1.2 software (Ronquist and Huelsenbeck 2003; Huelsenbeck *et al.* 2001; Altekar *et al.* 2004).

The following settings were used in our MCMC analysis: two runs, 750,000 generations; number of chains = 240; sample frequency = 250; 240 processors were used in parallel. The evolutionary model used was the WAG model that allows for 20 states. Rates were set to “Invgamma,” with the gamma shape parameter uniformly distributed on the interval (0.00, 200.00). The proportion of invariable sites was also uniformly distributed on the interval (0.00, 1.00). All topologies were equally probable, and branch lengths were unconstrained.

### Rate testing

To address the question of whether certain orthologous protein-encoding genes present in *Enallagma* were evolving at different rates relative to those of other arthropods, branch length rate tests were conducted with each *Enallagma* gene in our dataset. Using PAML (Yang 2007), we generated two models for each protein, one that assumed a global clock across all species and the other that fixed the rate of evolution of each *Enallagma* protein to a local clock while keeping the rest of the species evolutionary rates confined to a global clock. In this manner, we generate two likelihood estimates (one for each model) for these proposed modes of evolution of a particular protein. To that extent, a likelihood ratio test was performed between the null model (global clock) and alternative model (local clock).

$$D=-2^{*}({\mathrm{lnL}}_{G}-{\mathrm{lnL}}_{L})$$

Where *D* is the test statistic, *lnL_G_* is the log likelihood of the global clock model, and *lnL_L_* is the log likelihood of the local clock model. The probability distribution of the test statistic, *D*, can be approximated by the chi-squared distribution, where the degree of freedom of the distribution is equal to the number of free parameters of the global model minus the number of free parameters of the local model, which for our purposes, will be 1. (Parameters of the local model = 11 parameters of global model = 10.) Once a raw probability for each likelihood ratio was calculated, we performed Bonferroni corrections to determine significance.

### GO annotation

The complete set of all *Enallagma hageni* proteins was queried against a local NCBI “nonredundant” (nr) protein database (obtained October 14, 2011) using mpiBLAST. The output was saved in .xls format (-m 7–output-search-stats), which was then analyzed using Blast2GO without graphical interface (B2G4PIPE) and a local B2G database (Conesa *et al.* 2005).

We examined GO term distributions for three partitions of our dataset. First, we derived the distributions of 3^rd^- and 4^th^-level GO term hierarchies for the complete dataset of *Enallagma* proteins. The hierarchical system of gene ontology is represented as a directed acyclic graph in which parent-child relationships describe specific GO terms. That is, parent terms are less specific in their description of a biological function than are their respective child terms. This leads to “levels” within the Gene Ontology structure, with the 1^st^ level containing the broadest categories: biological processes, cellular components, and molecular function. An individual gene may then have many parents and many levels of categorization before reaching the 1^st^ level (Yon Rhee *et al.* 2008). Second, using *Drosophila melanogaster* as a background dataset, we determined those *Enallagma* genes that were enriched by a hypergeometric distribution test and corrected for multiple tests with false discovery rate (FDR) under dependency (Groppe 2012; Benjamini and Yekutieli 2001). Finally, we evaluated those *Enallagma* genes that were shown to have undergone either accelerated or reduced rates of evolution, per the branch length rate tests. These genes were examined for their overall GO 3^rd^- and 4^th^-level profiles and analyzed to determine if any gene was enriched. Enrichment was determined by setting all *Enallagma* genes as a background and using the hypergeometric test with FDR correction mentioned above.

We constructed a hash for each of the 3 partitions, using the annotations from the Blast2GO pipeline. Each gene and that gene’s associated GO accession terms made up the key:value relationship, which was then imported into the WeGO web-based program in order to sort the data by GO term hierarchy (Ye *et al.* 2006).

## Results

### Transcriptome assembly

After assembly, we obtained 31,662 contigs made up of 13,191,394 nucleotides. Of these contigs, 1656 were singletons (5.23%). Median coverage was 25 reads/contig (mean = 179.71 reads/contig; SD = 746.27), and median contig length was 355 bases (mean = 416.6; SD = 429.7). With singletons excluded, the dataset was reduced to 29,996 contigs. Of these, median coverage was 26 reads/contig (mean coverage = 173.73 reads/contig; SD = 677.99), and median contig length was 406 bases/contig (mean contig length = 439.7; SD = 429.9). The largest contig in the dataset was composed of 3036 nucleotides. The assembled transcriptome contained an AT bias at 59.86% and GC at 40.13%, and 0.01% was labeled “N.” CpG sites occurred at 2.69% of the transcriptome. (see Figure S2 and Table S5 for assembly details).

### Translated proteins

Translation of the *Enallagma* contigs yielded 14,813 individual open reading frames consisting of 1,621,208 amino acids (singletons not included). Mean length was 109 amino acids. Shortest and longest protein sequences were composed of 19 amino acids and 735 amino acids, respectively (see Figure S3).

### Orthologs

The one-to-one, reciprocal best method of elucidating orthologous proteins generated 634 orthologs across the 11 species in the study. The *Enallagma* orthologs themselves contained 108,866 amino acids with a mean length of 171 amino acids, and shortest and longest sequence length of 46 amino acids and 413 amino acids, respectively (see Table S3 for ortholog groups.)

### GO annotation

**Figure 1:**
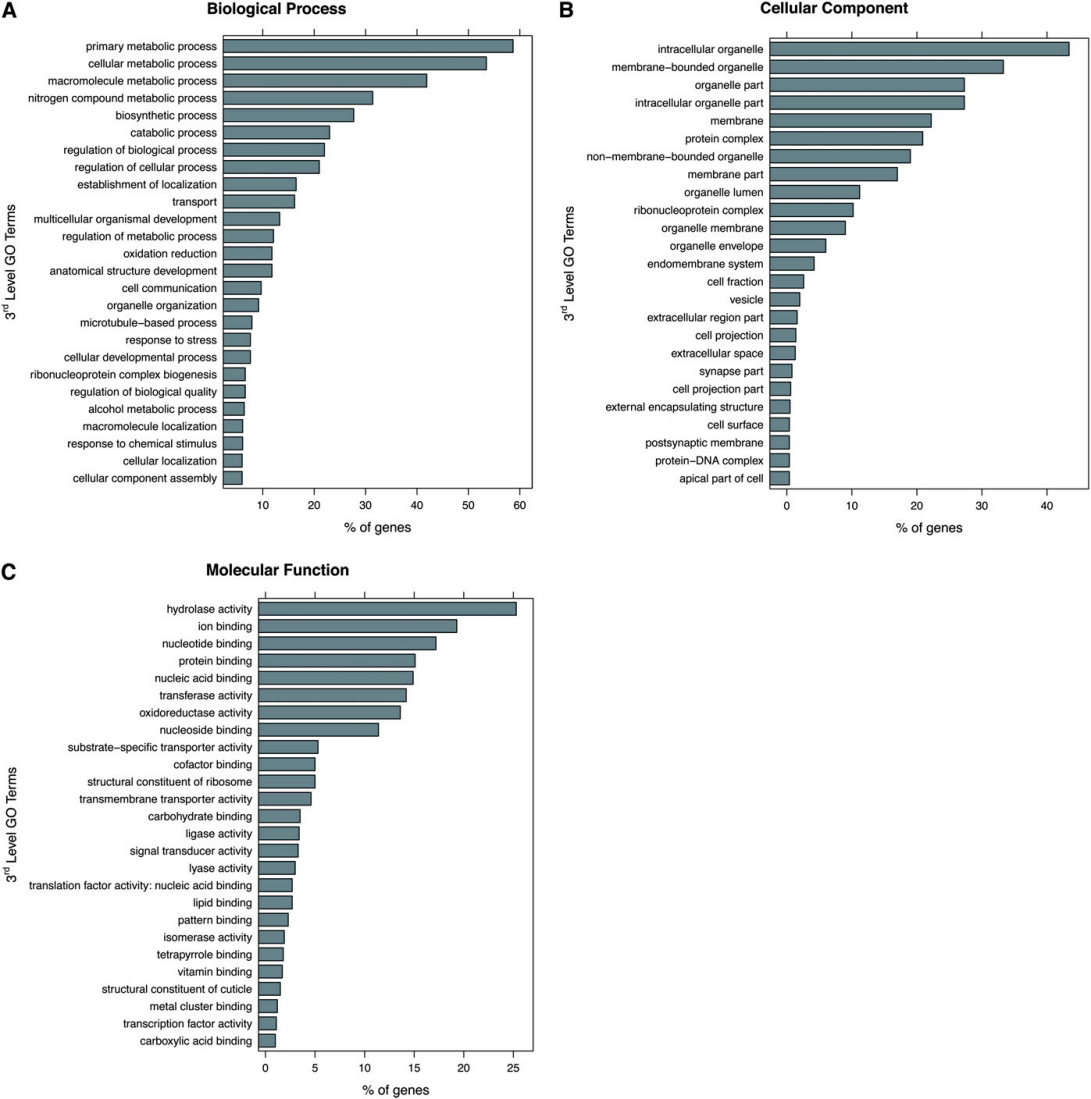
3^rd^-level GO term distributions for all annotated *Enallagma* genes. GO term distributions were plotted for each of the three 1^st^-level categories. The full dataset mapped to 404 unique GO terms at the 3^rd^ level. Shown are the top 25 terms in each of the broadest, 1^st^-level categories: (A) Biological Process, (B) Cellular Component, and (C) Molecular Function.

**Figure 2:**
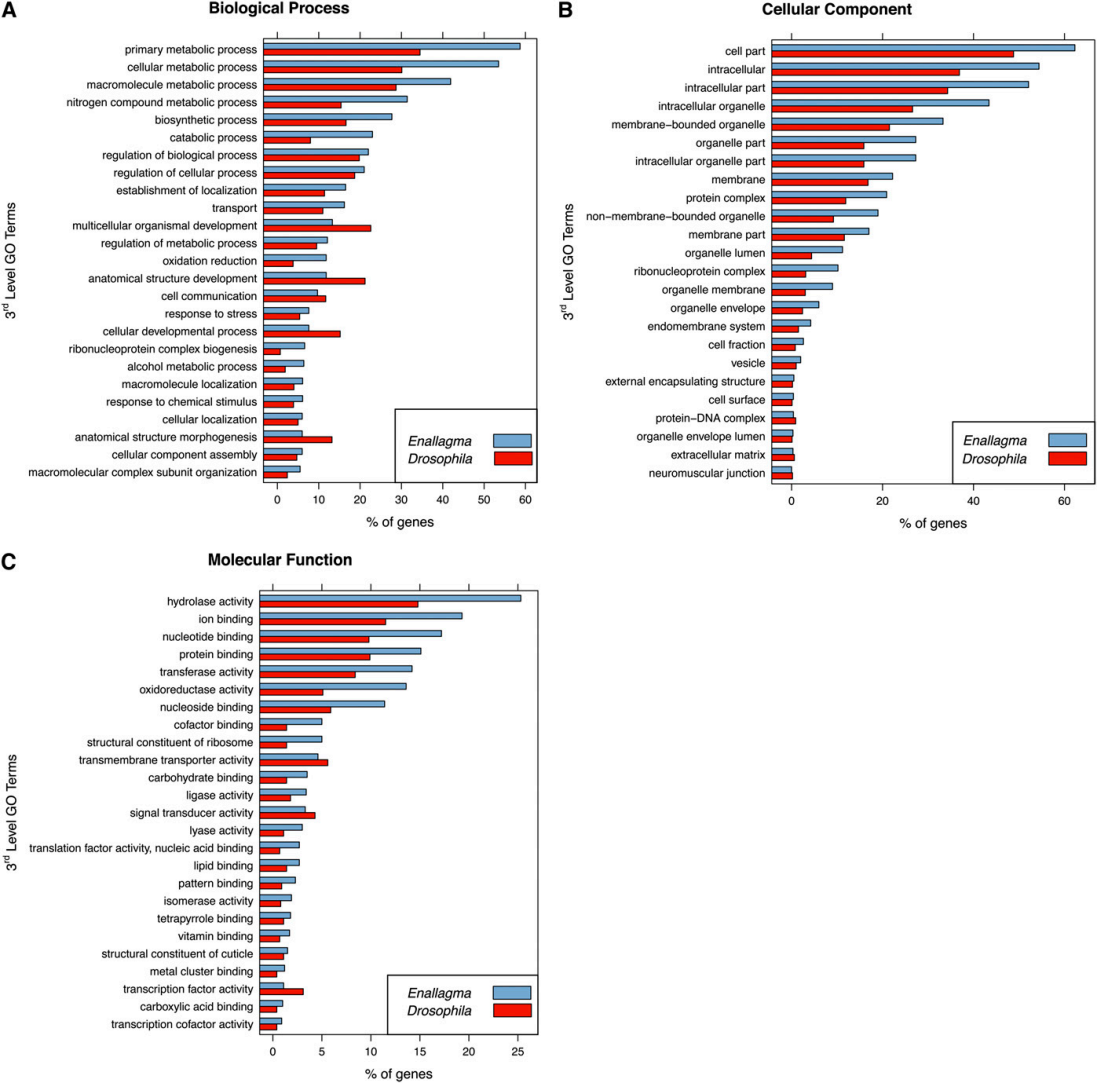
Enrichment or reduction of *Enallagma* GO terms relative to annotated *Drosophila melanogaster* genes. Using *D. melanogaster* as a background set, hypergeometric distribution tests were performed to identify *Enallagma* genes that were enriched or diminished. The background set consisted of 13,127 *D. melanogaster* annotated genes and was queried by 3986 *Enallagma* genes. We discovered 1080 unique enriched or diminished terms. (A) Biological Process, (B) Cellular Component, and (C) Molecular Function are the top 25 most significant results.

**Figure 3:**
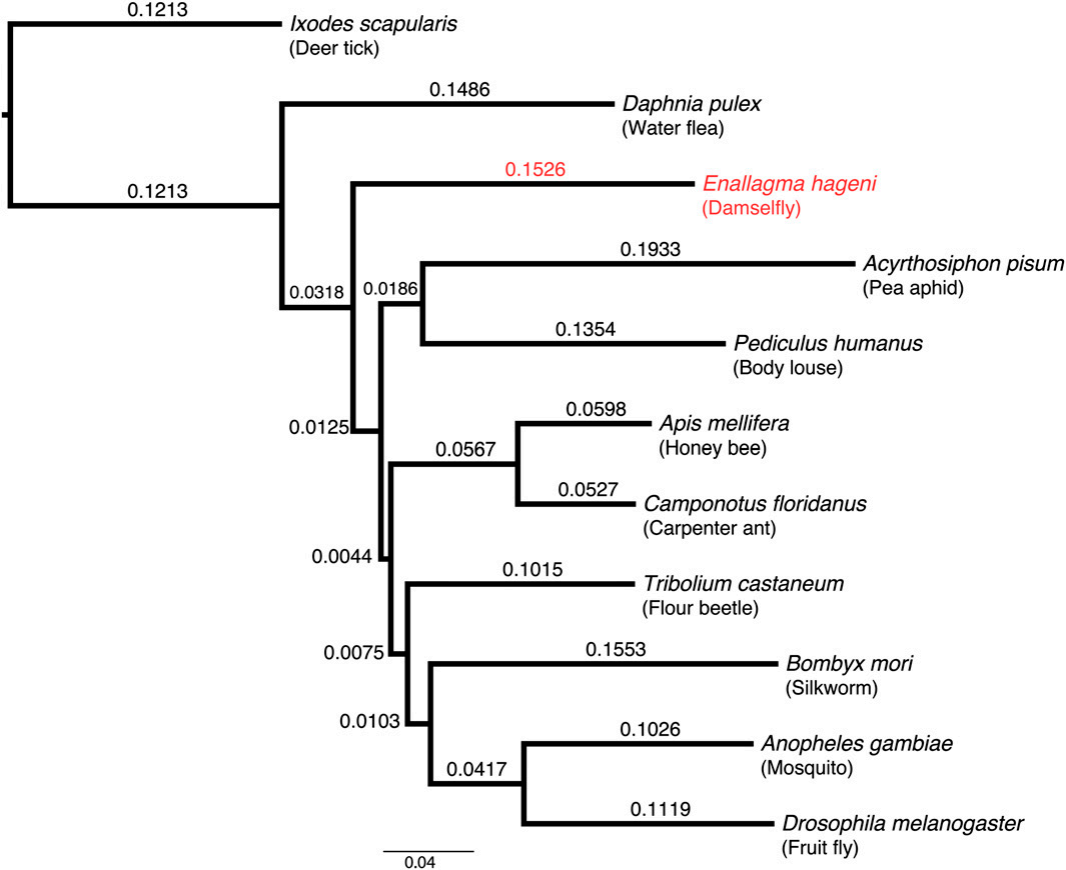
Arthropod phylogram. Eleven taxa and 27,594 amino acid positions were used in the analysis. Branch lengths are labeled, and posterior probabilities at each branching node are 1.0.

Our annotation methodology mapped 3998 *Enallagma* genes to at least one GO term, using BLAST2GO and the NCBI “nr” database. There were 24,439 total GO terms mapped to those 3998 genes, with 3812 of the GO terms being unique. The mean mapping was 6.1 GO terms/gene with a minimum and maximum mapping of 1 and 78 GO terms per gene, respectively. Using 3^rd^- and 4^th^-level GO term distributions, we mapped our dataset to 404 GO terms across 3 ontologies for 3^rd^-level terms (cellular component, biological process and molecular function) and 1463 terms across 3 ontologies for 4^th^-level terms. (Figures 2 and 3). At the 3^rd^ level of the hierarchy, the top GO terms represented were (1) biological processes: 58.7% of the genes were mapped to “primary metabolic processes” (GO:0044238), 53.5% of genes to “cellular metabolic processes” (GO:0044237), and 41.9% to “macromolecule metabolic processes” (GO:0043170); (2) cellular components: 43.4% to “intracellular organelles” (GO:0043229), 33.3% to “membrane-bound organelles” (GO:0043227), and 27.3% to “organelle parts” (GO:0044422); and (3) molecular function: 25.3% to “hydrolase activity” (GO:0016787), 19.3% to “ion binding” (GO:0043167), and 17.2% to “nucleotide binding” (GO:0000166). See Figure 1 for 3^rd^-level distribution. See Figure S4 for 4^th^-level distributions.

To look for enriched or diminished GO terms, we then compared the *Enallagma* GO annotations to *Drosophila melanogaster* GO annotations. We queried 3986 annotated *Enallagma* genes against 13,127 annotated *Drosophila* genes and found that 1080 unique (1089 total) *Enallagma* GO terms were enriched or diminished. Described in terms of the GO hierarchy, we discovered 33 2^nd^-level GO terms and 161 3^rd^-level GO terms.

Some of these enriched 3^rd^-level GO annotations included hydrolase activity (GO:16787), ion and nucleotide binding (GO:43167 and GO:0000166), and primary metabolic processes (GO:44238). Examples of diminished GO terms included anatomical structural development (GO:48856) and protein-DNA complex (GO:32993).

Additionally, we mapped 488 genes within the orthologous protein-encoding set to 1669 GO IDs, 691 of these GO IDs being unique (Figure 2); for the gene ID, GO ID, and gene product/function see Table S4.)

### Phylogenetics

After concatenating the 634 orthologous genes, the resulting multi-way alignment contained 182,478 amino acid positions. This alignment was then filtered with Gblocks, using the default parameters that do not allow for gaps at any position in the matrix, resulting in an ungapped alignment of 27,594 amino acid positions (15.1% of the original data). This ungapped matrix was then analyzed using MrBayes software with settings described in *Material and Methods*.

We removed 50 samples of burn-in after each MCMC run, therefore sampling from the posterior 2952 times for each of the two runs. Each of the two MCMC analyses took 224,340 seconds (62.3 hours) and 227,756 seconds (63.3 hours) to complete, respectively. The plotted phylogram, based on the consensus tree data of the MCMC runs, is shown in Figure 3.

*Ixodes scapularis* (class Arachnida) was chosen as the out group, and the tree was rooted upon it. The posterior probability for each node in the tree was 1.0.

Trace plots of the MCMC analysis and Gelman convergence plots are shown in Figure S5 and Figure S6.

### Rate testing

The branch length test indicated that 439 of the 634 (69.2%) orthologs fit a local clock model better and were therefore deduced to be evolving at a rate that varied relative to that gene’s orthologs (raw *P* < 0.05). However, a Bonferroni correction for multiple tests, (*P* < 0.05/634 = 0.0000788) reduced that set and yielded 169 genes which were shown to be evolving at significantly different rates in *Enallagma*. Of these 169 genes, 29 genes were shown to be evolving at an accelerated rate, while the remaining 140 genes were determined to be evolving at a reduced rate. We successfully mapped 37 of these genes to at least one GO term. In the accelerated case, 4 of the 29 genes were mapped to 17 GO terms, while in the decreased case, 33 of the 140 genes mapped to 105 GO terms. Of those 37 genes that we were able to annotate, no significant enrichment was noted by using the hypergeometric test (*P* < 0.05), relative to the background set of all *Enallagma* GO annotations. Table S1 shows the 4 accelerated genes and their gene products. These include *Nol10* (nucleolar protein), *Art7* (protein arginine N-methyltransferase), *Rrp45* (RNA processing), and *Uba3* (ubiquitin-like protein). Figure S7 and Figure S8 show 3^rd^- and 4^th^-level distributions of the decreased rate genes (see also Table S2.)

## Discussion

At the level of resolution we used to examine (other species within Arthropoda which had assembled transcriptomes), our phylogenetic analysis of *Enallagma* and the compared arthropods appears congruent to that of other current studies and reviews (Meusemann *et al.* 2010; Ishiwata *et al.* 2011; Trautwein *et al.* 2012).

Our hypergeometric tests of the accelerated and decreased rates of proteins’ GO annotations, relative to the background set of all genes we were able to annotate, indicated no significant enrichments (*P* < 0.05 raw, FDR corrections). Nevertheless, the GO term distributions of the altered rate genes were shown to similarly represent the distributions of the overall dataset. For example, the top three GO terms represented by both the biological processes and cellular component 3^rd^-level domains were the same. In the case of biological processes, we saw the terms “primary metabolic process,” “cellular metabolic process,” and “macromolecule metabolic process” encompassing the top three positions, while the top three terms in the domain of cellular component were “intracellular organelle,” “membrane-bounded organelle,” and “intracellular organelle part.” However, there were some deviations from that, especially in the molecular function domain. For example, the top two GO terms represented in the decelerated genes category, in the “Molecular Function” domain, were shown to be “nucleotide binding” and “nucleic acid binding,” whereas in the full set, the top two expressed GO terms for that same domain were “hydrolase activity” and “ion binding”.

One of the interesting ecological and evolutionary scenarios involving *Enallagma* is that various *Enallagma* lineages have adapted to living with predators by increasing their burst swimming speeds to increase their probability of escape during predator attacks (McPeek *et al.* 1996; McPeek 1999; McPeek 2000). In agreement with this, we annotated genes involved in muscle mass increase and differentiation (GO:0003012) and genes with roles in arginine kinase (GO:0004054) and arginine methylation [accelerated (see Table S1); GO:0019918], which has been shown to partially responsible for the observed rapid movements of the damselflies (McPeek 1999; McPeek 2000).

Another issue worth noting is that analysis by short read sequencing in transcriptome assembly relies on the use of reads typically 35–250 bp in length (Mardis 2008; Harismendy *et al.* 2009). Our annotation methodology mapped 3998 *Enallagma* genes to at least one associated GO term. While this number represents less than 30% of the genes in our dataset associating with a GO term, it should be noted that small contigs, like those generated in 454 sequencing, can be difficult to successfully map to GO terms and that mapping success increases successively with read size. (Novaes *et al.* 2008; Meyer *et al.* 2009).

In summary, we have generated a draft functional annotation of nearly 4000 genes in the transcriptome of *Enallagma hageni*, which to our knowledge is the first examined and annotated transcriptome of any palaeopteran in the literature. We examined the rate at which *E. hageni* proteins are evolving and found 169 genes which better fit the hypothesis of having an altered evolutionary history, relative to other genes in its transcriptome. We examined the distributions of GO terms for each of three classes of our data: the whole annotated transcriptome, the transcriptome with *D. melanogaster* as a background, and the set of altered genes with all *Enallagma* genes as a background. Of those, we additionally deduced which annotations are enriched or diminished through the use of hypergeometric distribution testing. Finally, we have produced a strongly supported phylogenetic analysis that in turn further strengthens support for the position of Odonata in the Arthropoda tree.

## Supplementary Material

Supporting Information
