# Supplementary material for: Functional Annotation and Comparative Analysis of a Zygopteran Transcriptome
Source: G3 (Bethesda). 2013 Apr 1;3(4):763–70. doi: 10.1534/g3.113.005637 (PMC3618363; doi:10.1534/g3.113.005637)
Supplement: Supporting Information [file supp_3_4_763__index.html]

Functional Annotation and Comparative Analysis of a Zygopteran Transcriptome — Supporting Information 

# Functional Annotation and Comparative Analysis of a Zygopteran Transcriptome

## Supporting Information for Shanku, McPeek, and Kern, 2013

**Files in this Data Supplement:**

- Supporting Information - Figures S1-S8 and Tables S1-S5 (PDF, 572 KB)
- Figure S1 - Transcriptome content (PDF, 69 KB)
- Figure S2 - Nucleotide profile of assembled *Enallagma* contigs (PDF, 65 KB)
- Figure S3 - Amino acid profile (PDF, 69 KB)
- Figure S4 - Fourth level GO term distributions for all annotated *Enallagma* genes (PDF, 116 KB)
- Figure S5 - Trace and density plots of the posterior probability of the phylogenetic analysis (PDF, 146 KB)
- Figure S6 - Gelman and Rubin convergence plot of the MCMC analysis (PDF, 56 KB)
- Figure S7 - Third level GO term distribution for decreased rate genes (PDF, 111 KB)
- Figure S8 - Fourth level GO term distribution for decreased rate genes (PDF, 106 KB)
- Table S1 - Accelerated genes and their gene products (PDF, 67 KB)
- Table S2 - Decreased rate annotations (.txt, 2 KB)
- Table S3 - Gene ID's (.csv, 117 KB)
- Table S4 - Annotated Orthologs (.txt, 91 KB)
- Table S5 - Newbler assembler (v.2.3) parameters (.txt, 1 KB)
