# Supplementary material for: Functional Annotation and Comparative Analysis of a Zygopteran Transcriptome
Source: G3 (Bethesda). 2013 Apr 1;3(4):763–70. doi: 10.1534/g3.113.005637 (PMC3618363; doi:10.1534/g3.113.005637)
Supplement: Supporting Information [file supp_g3.113.005637_005637SI.pdf]

G3: Genes | Genomes | Genetics

Functional annotation and comparative analysis of a Zygopteran transcriptome

Alexander G. Shanku[1], Mark A. McPeek[2], and Andrew D. Kern[3]

1. Rutgers University
2. Dartmouth College
3. Rutgers University / Human Genetics Institute of New Jersey
4.

DOI: 10.1534/g3.113.005637

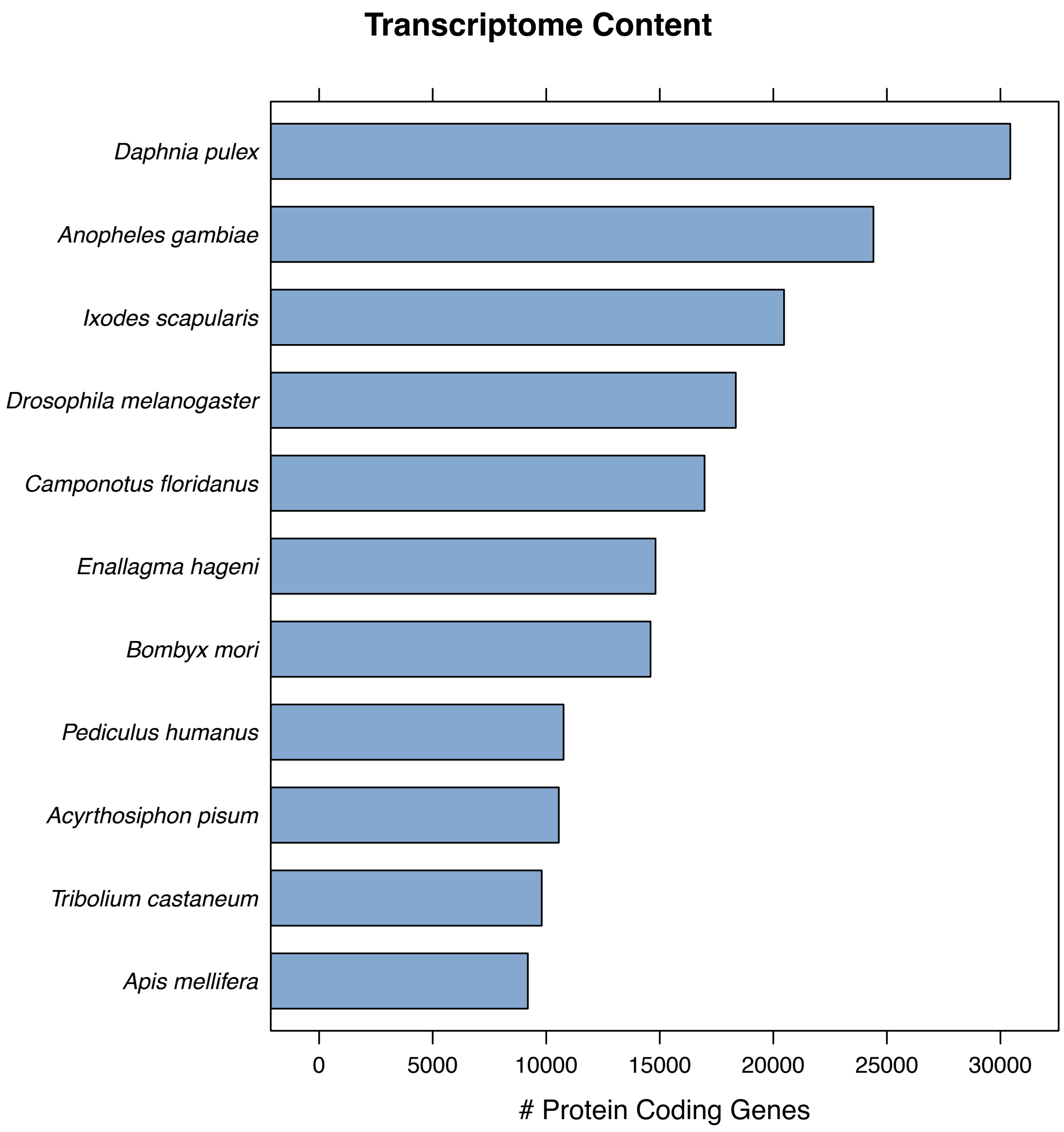

Figure S1 Transcriptome content. The number of protein coding genes of the 11 species used in our analysis is shown. The *Enallagma hageni* transcriptome possesses 14,813 protein coding genes.

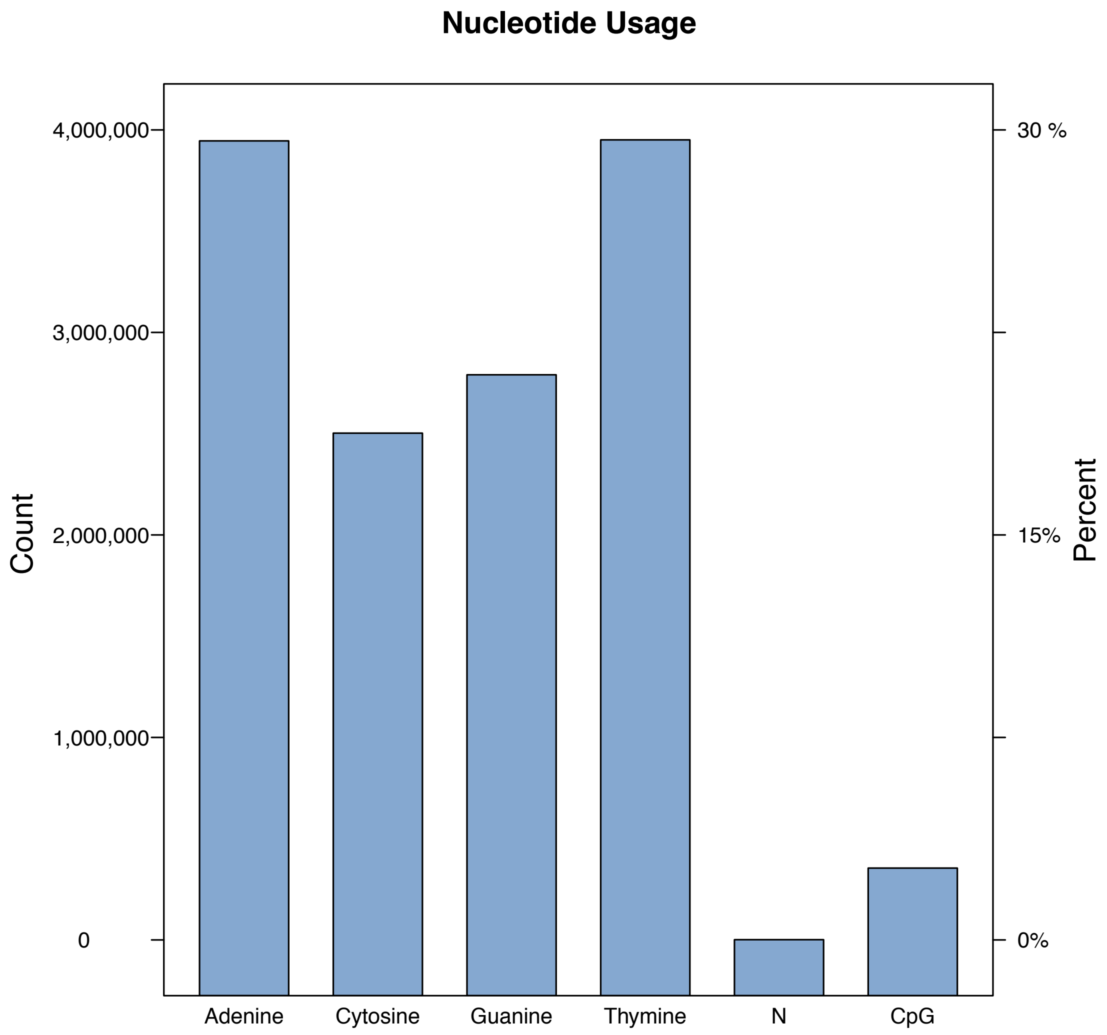

Figure S2 Nucleotide profile of assembled *Enallagma* contigs. The assembled *E. hageni* transcriptome is comprised of 13,191,394 nucleotides. An AT bias is observed (59.86% AT, 40.13% GC, 0.01%N) and CpG sites occurred in 2.69% of the assembled transcriptome.

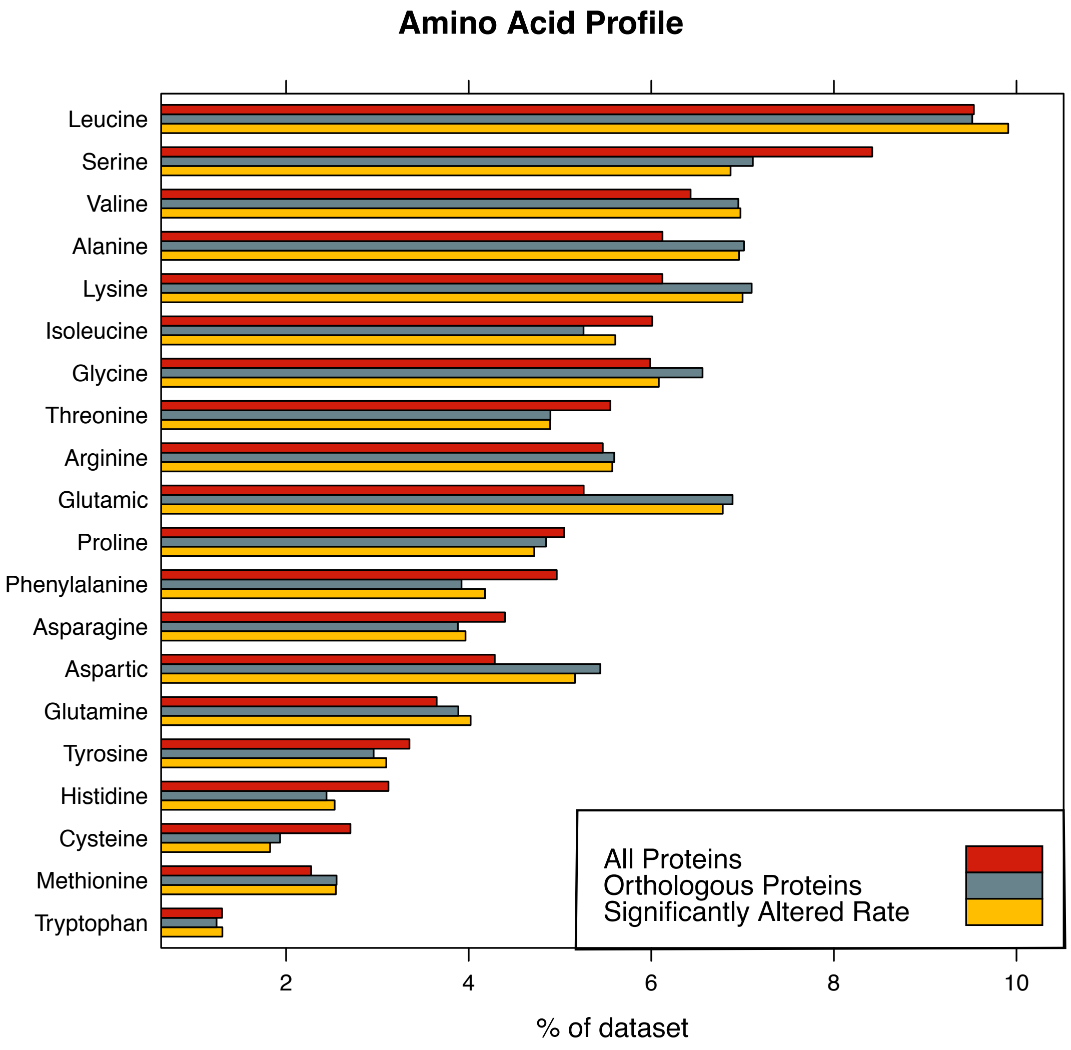

Figure S3 Amino acid profile. The amino acid profiles of three groups of translated *Enallagma* proteins are presented. The profile of all 1,621,208 amino acids comprising the 14,813 protein coding genes is shown in red. The 634 proteins orthologous across all 11 arthropod species in this study are indicated in grey and the 169 genes shown to have at an altered rate are shown in yellow.

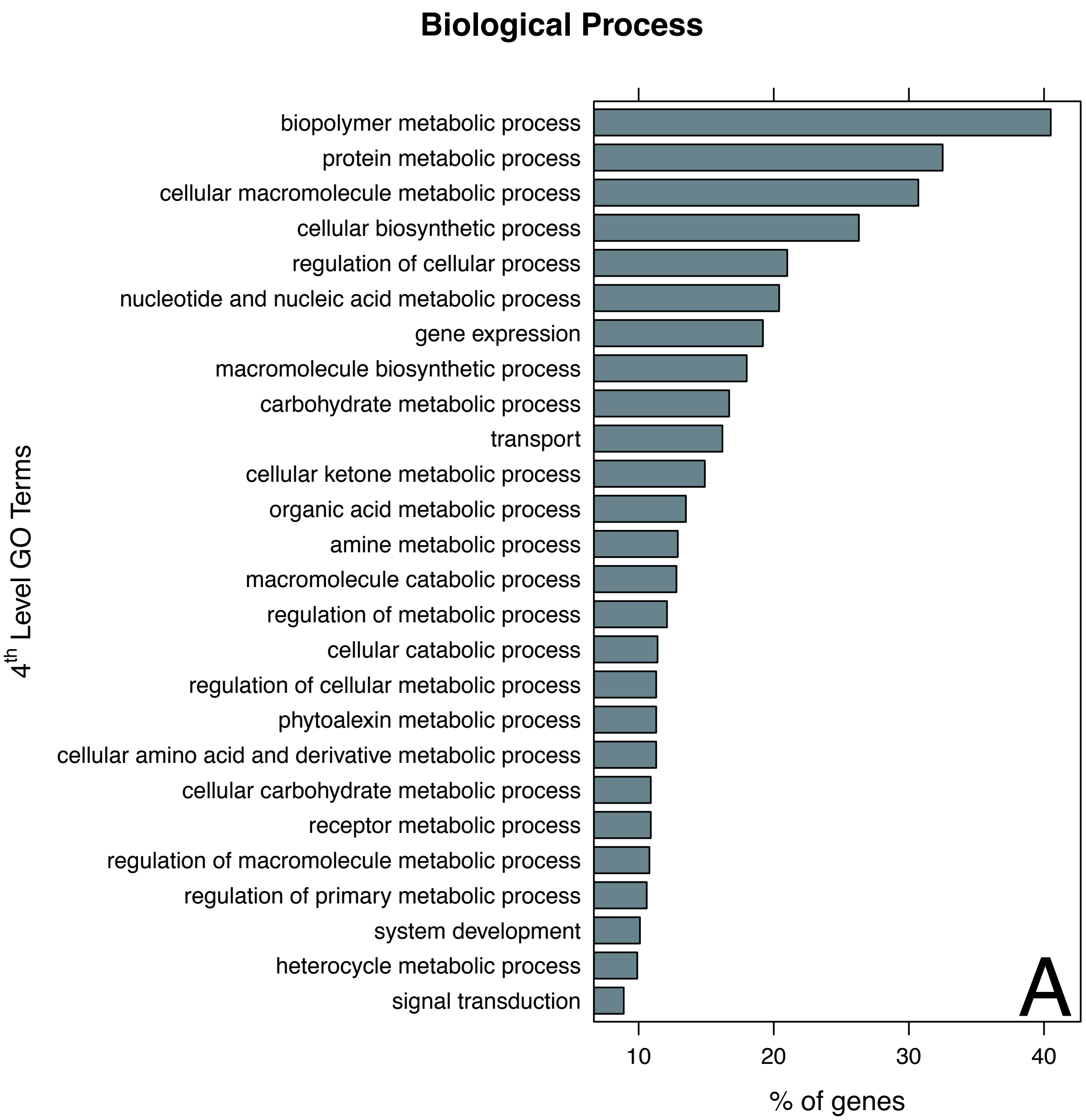

Biological Process
biopolymer metabolic process
protein metabolic process
cellular macromolecule metabolic process
cellular biosynthetic process
regulation of cellular process
nucleotide and nucleic acid metabolic process
gene expression
macromolecule biosynthetic process
carbohydrate metabolic process
transport
cellular ketone metabolic process
organic acid metabolic process
amine metabolic process
macromolecule catabolic process
regulation of metabolic process
cellular catabolic process
regulation of cellular metabolic process
phytoalexin metabolic process
cellular amino acid and derivative metabolic process
cellular carbohydrate metabolic process
receptor metabolic process
regulation of macromolecule metabolic process
regulation of primary metabolic process
system development
heterocycle metabolic process
signal transduction
4th Level GO Terms
10
20
30
40
% of genes
A

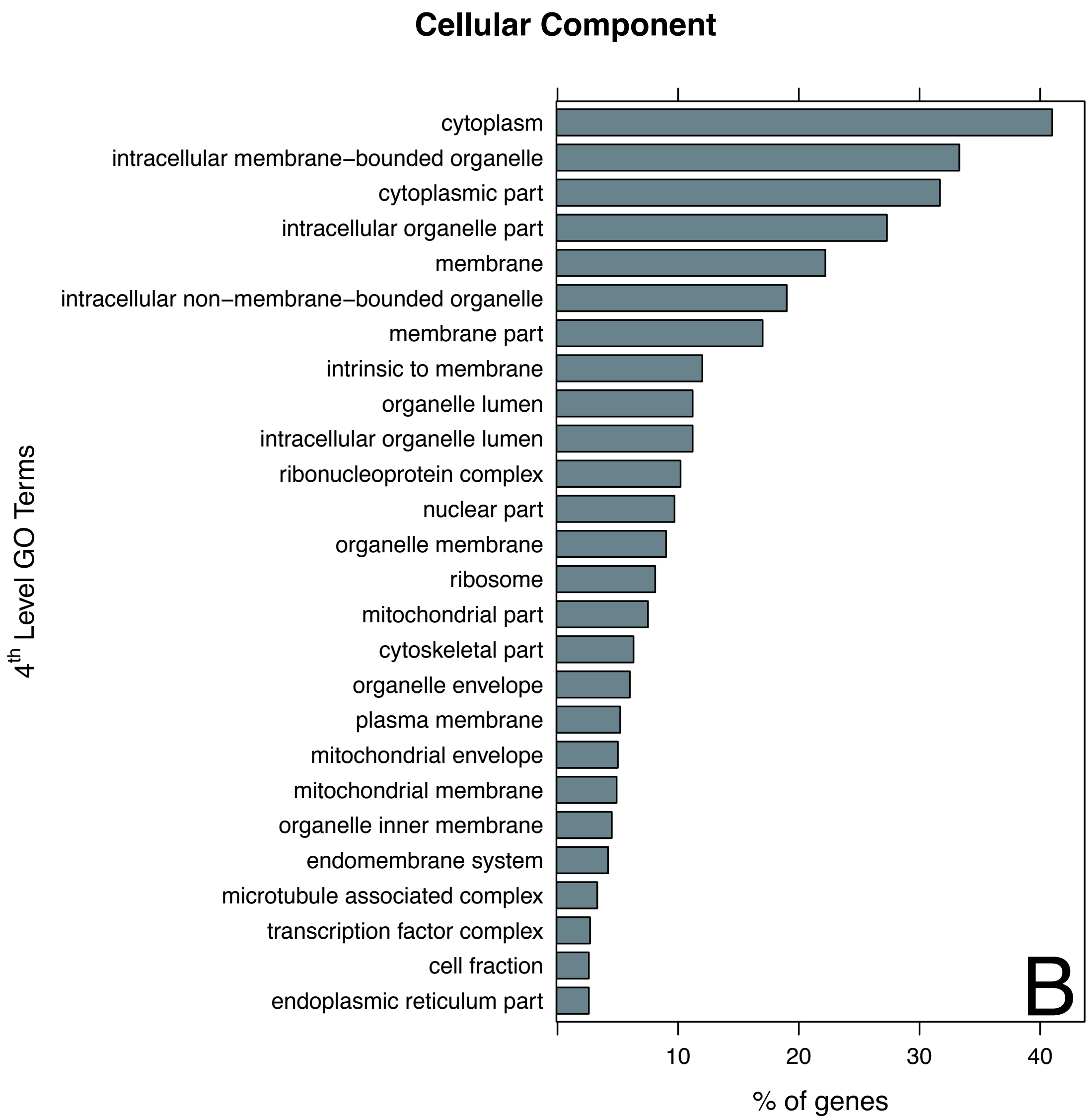
Cellular Component
cytoplasm
intracellular membrane−bounded organelle
cytoplasmic part
intracellular organelle part
membrane
intracellular non−membrane−bounded organelle
membrane part
intrinsic to membrane
organelle lumen
intracellular organelle lumen
ribonucleoprotein complex
nuclear part
organelle membrane
ribosome
mitochondrial part
cytoskeletal part
organelle envelope
plasma membrane
mitochondrial envelope
mitochondrial membrane
organelle inner membrane
endomembrane system
microtubule associated complex
transcription factor complex
cell fraction
endoplasmic reticulum part
4th Level GO Terms
10
20
30
40
% of genes
B

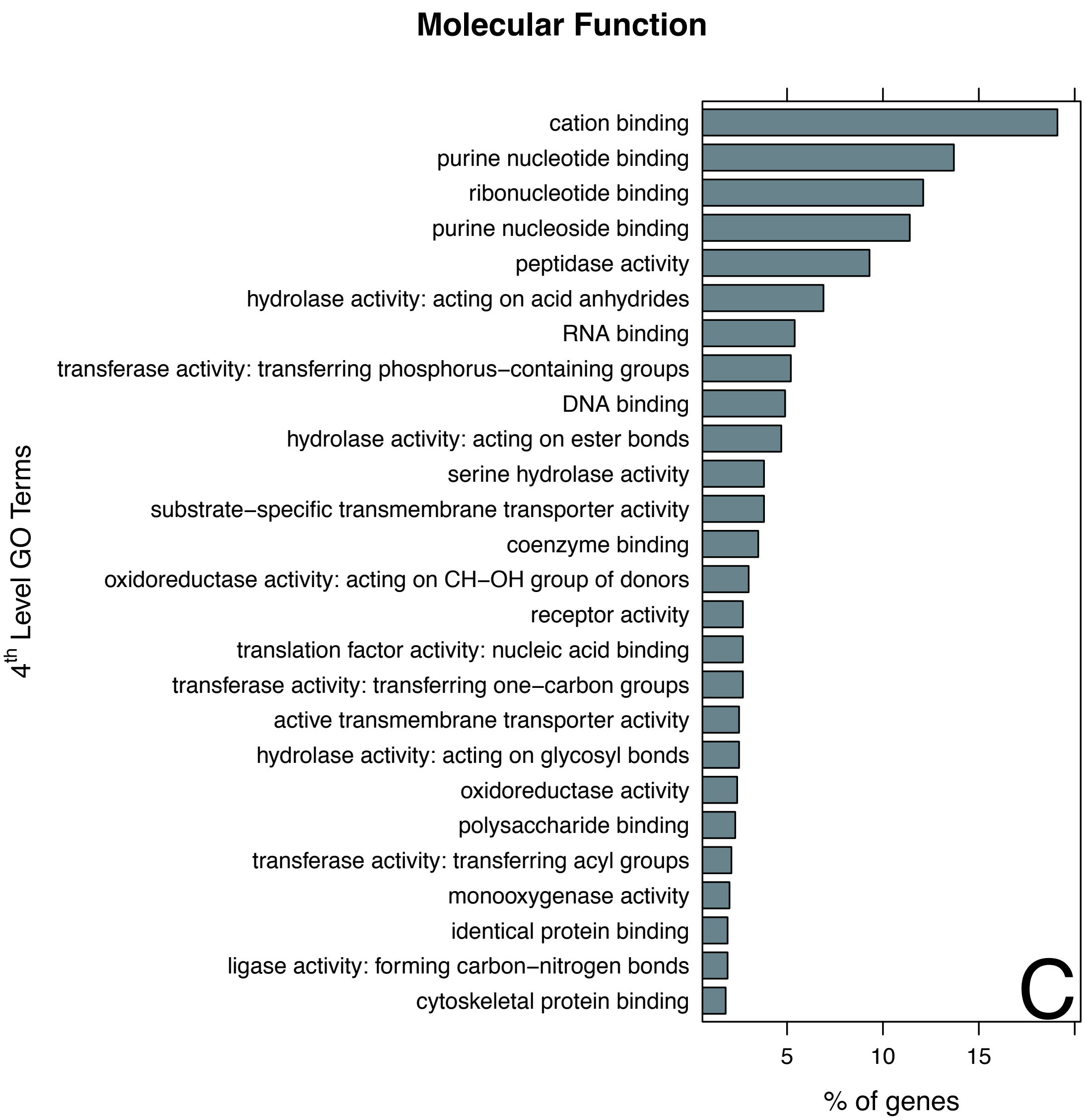

Figure S4 4th level GO term distributions for all annotated *Enallagma* genes. At the 4th level of the GO term hierarchy, we mapped the dataset of genes to 1463 GO terms across the 3 ontologies. Shown are the top 25 most significant results in each of the 1st level categories, A) biological processes, B) cellular components, and 3) molecular function.

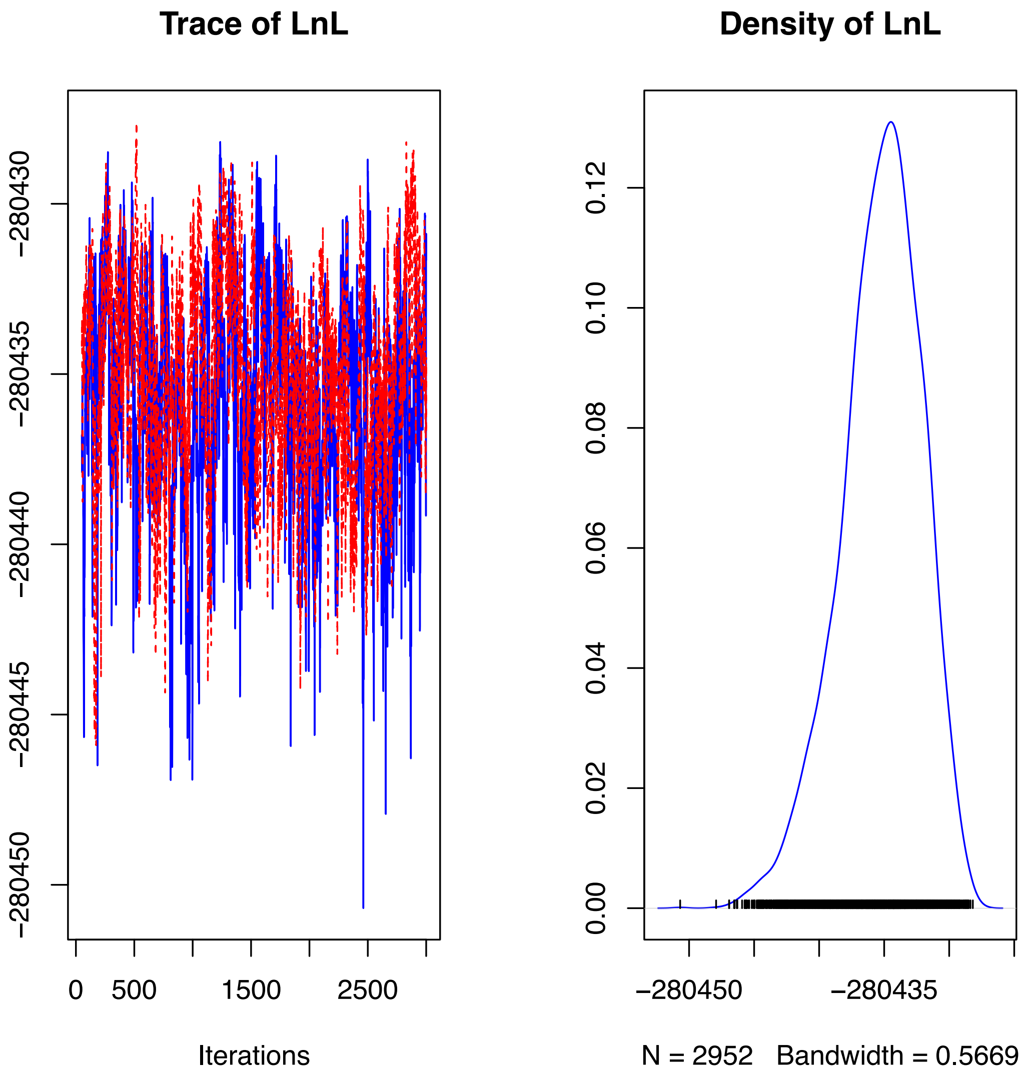

Figure S5 Trace and density plots of the posterior probability of the phylogenetic analysis. After thinning the samples of the posterior probability, we obtained 2952 draws from the posterior. Shown in (A) is the negative log-likelihood trace plot. In (B) we plot the density of the thinned posterior.

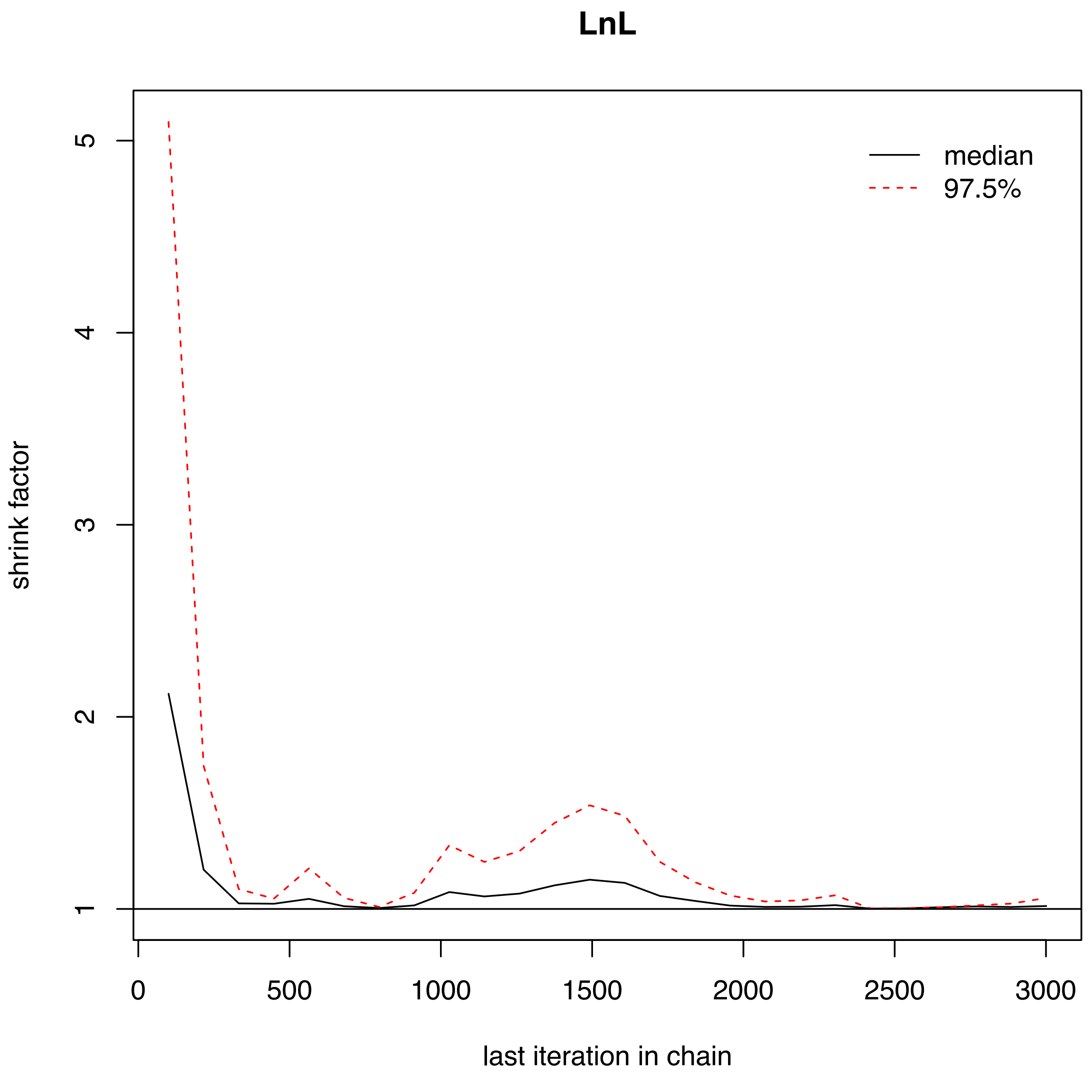

Figure S6 Gelman and Rubin convergence plot of the MCMC analysis. To test that our chains have converged to the stationary distribution, the thinned samples from both chains in the MCMC run are used to compute the Gelman and Rubin test for convergence. This test calculates the within-chain and between-chain variance and returns a potential scale reduction factor. If this factor is below ~1.25, it is another assurance that the stationary distribution has been reached.

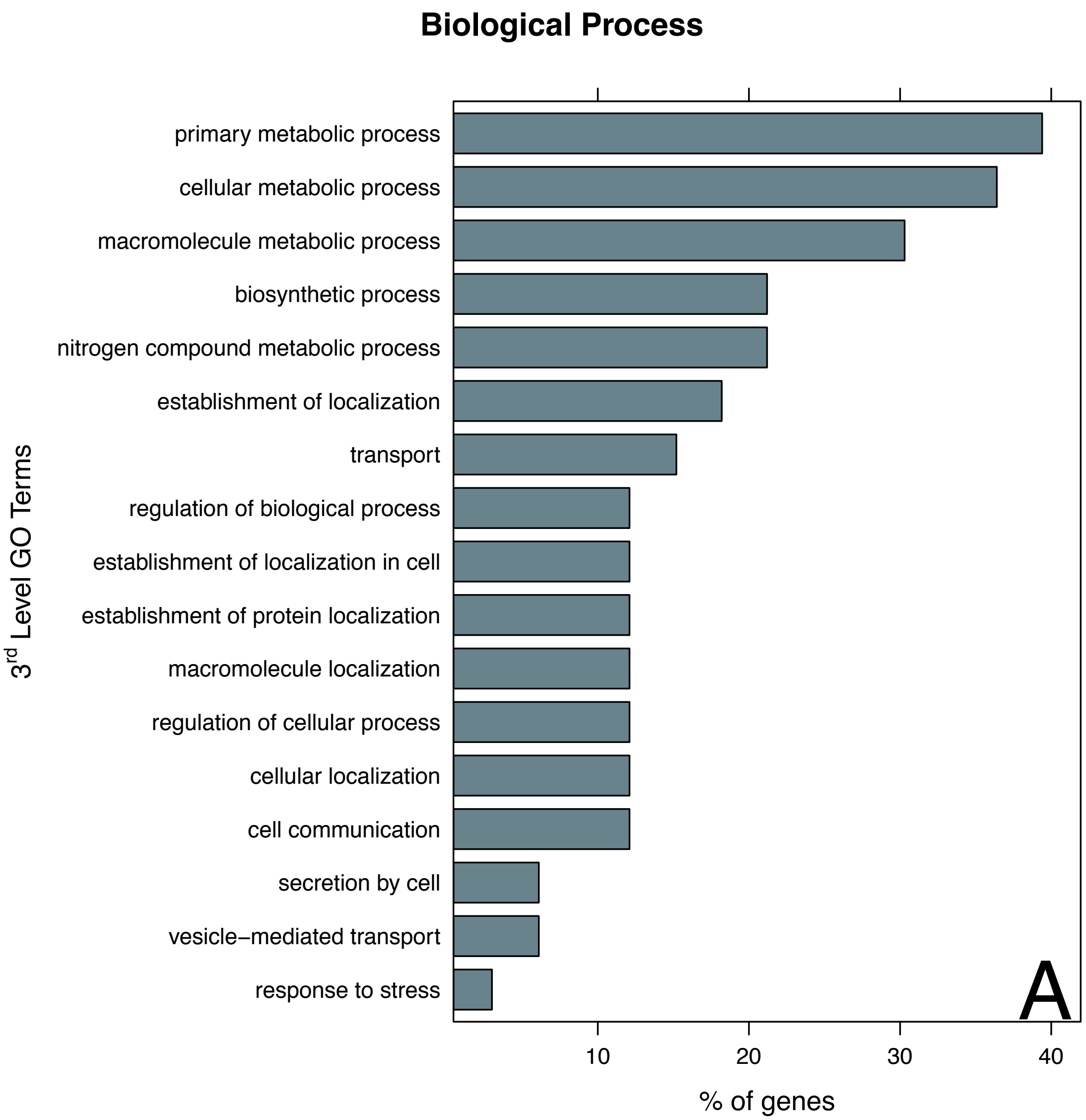
Biological Process
primary metabolic process
cellular metabolic process
macromolecule metabolic process
biosynthetic process
nitrogen compound metabolic process
establishment of localization
transport
regulation of biological process
establishment of localization in cell
establishment of protein localization
macromolecule localization
regulation of cellular process
cellular localization
cell communication
secretion by cell
vesicle–mediated transport
response to stress
A
3rd Level GO Terms
10
20
30
40
% of genes

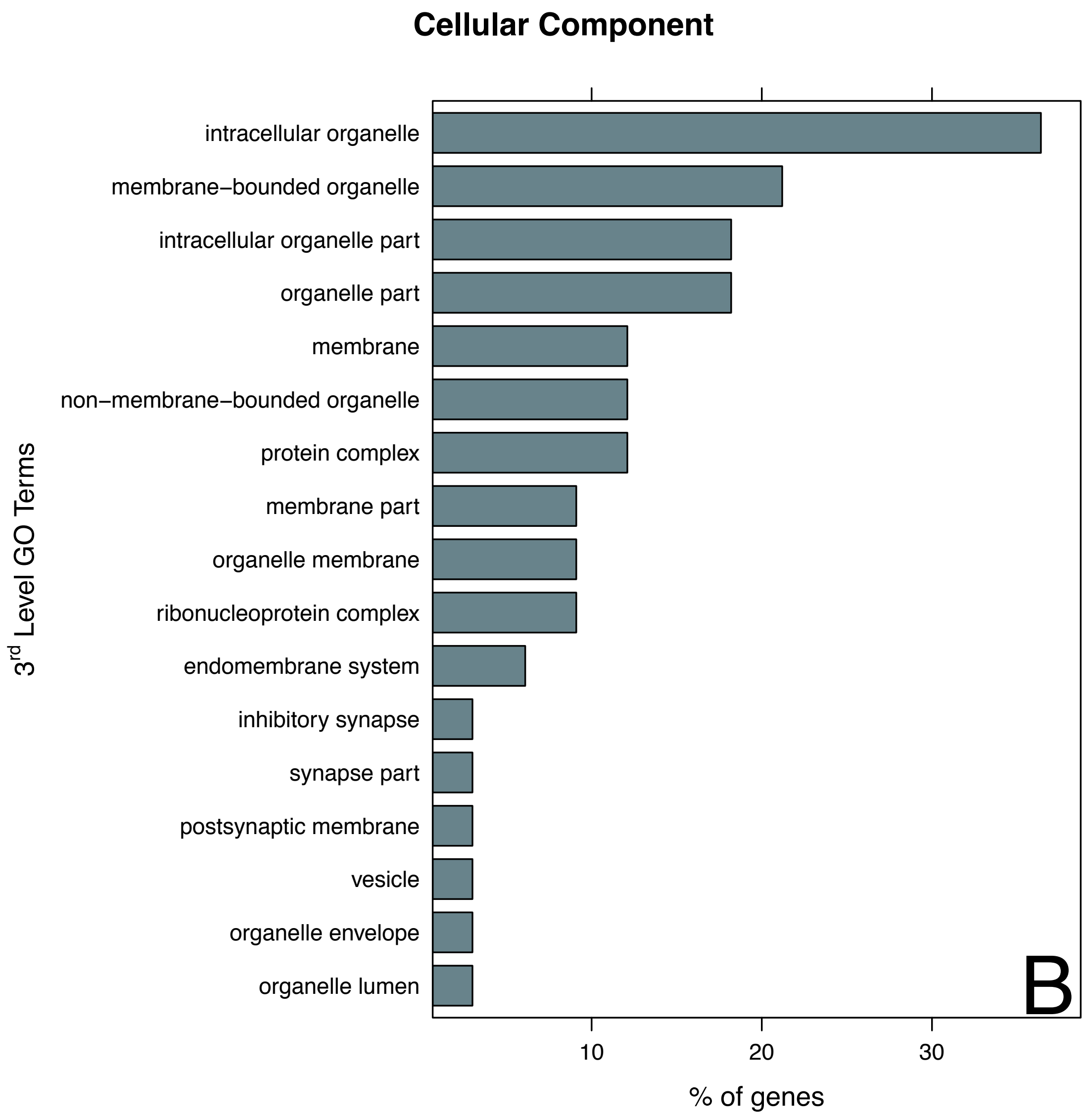
Cellular Component
intracellular organelle
membrane−bounded organelle
intracellular organelle part
organelle part
membrane
non−membrane−bounded organelle
protein complex
membrane part
organelle membrane
ribonucleoprotein complex
endomembrane system
inhibitory synapse
synapse part
postsynaptic membrane
vesicle
organelle envelope
organelle lumen
B
10
20
30
% of genes
3rd Level GO Terms

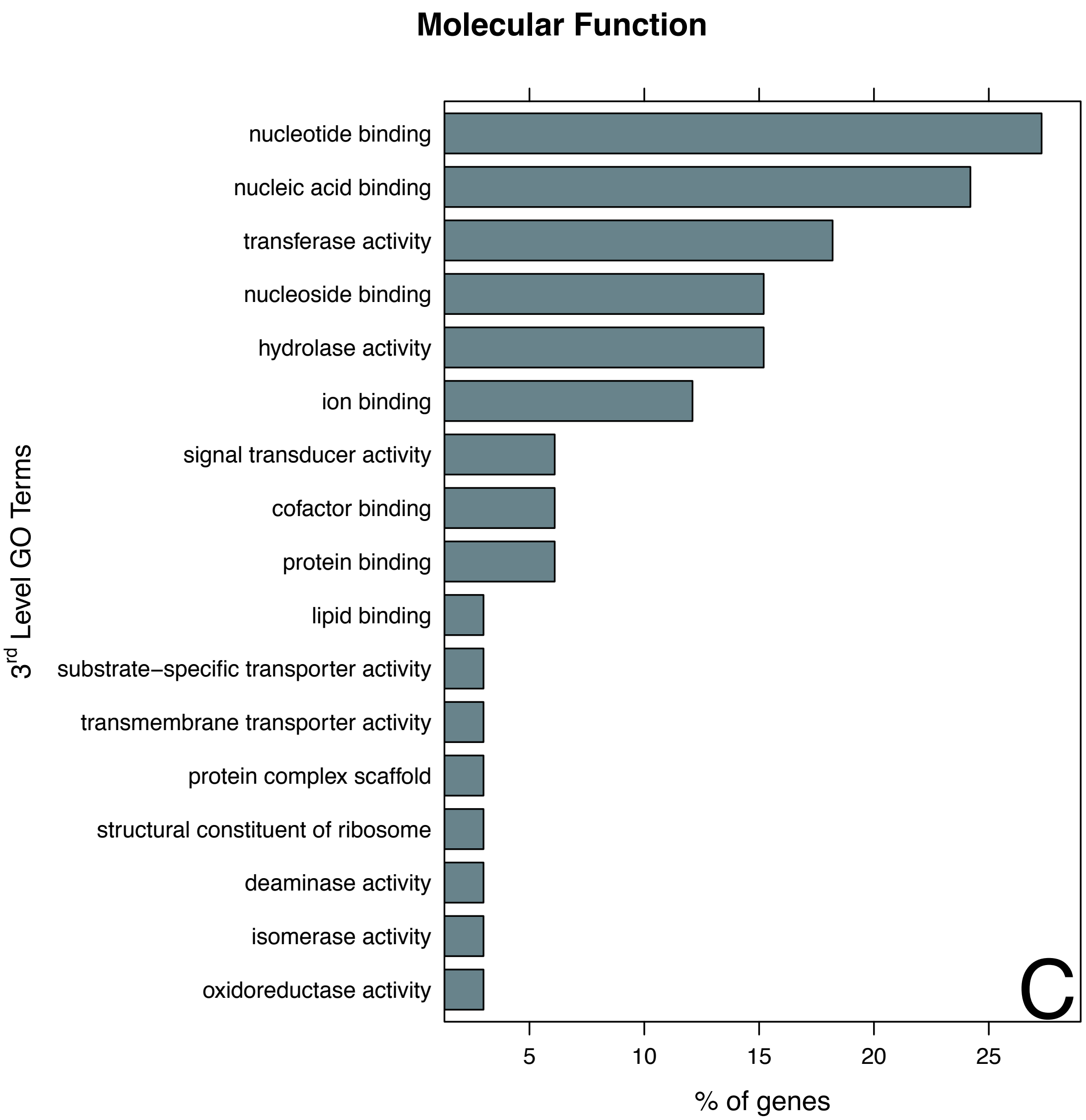

Figure S7 3rd level GO term distribution for decreased rate genes. Of the 140 *Enallagma hageni* genes which were shown to be evolving at either a diminished rate, per the branch length tests, we were able to map 33 of these genes to 105 GO terms. Shown here are the top 17 most significant of these terms across the three orthologies, (a),(b), and (c).

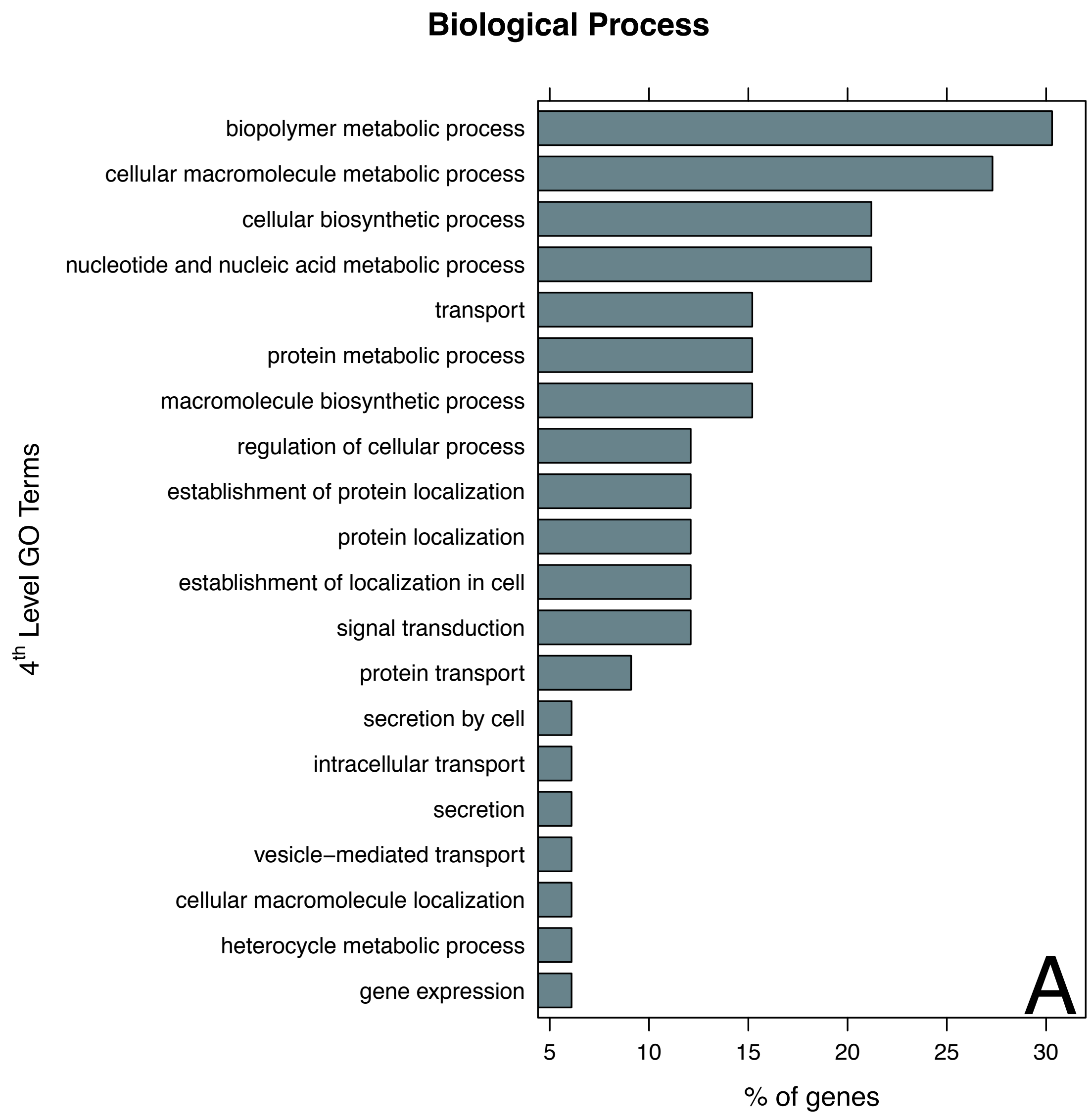

Biological Process
biopolymer metabolic process
cellular macromolecule metabolic process
cellular biosynthetic process
nucleotide and nucleic acid metabolic process
transport
protein metabolic process
macromolecule biosynthetic process
regulation of cellular process
establishment of protein localization
protein localization
establishment of localization in cell
signal transduction
protein transport
secretion by cell
intracellular transport
secretion
vesicle−mediated transport
cellular macromolecule localization
heterocycle metabolic process
gene expression
4th Level GO Terms
5
10
15
20
25
30
% of genes
A

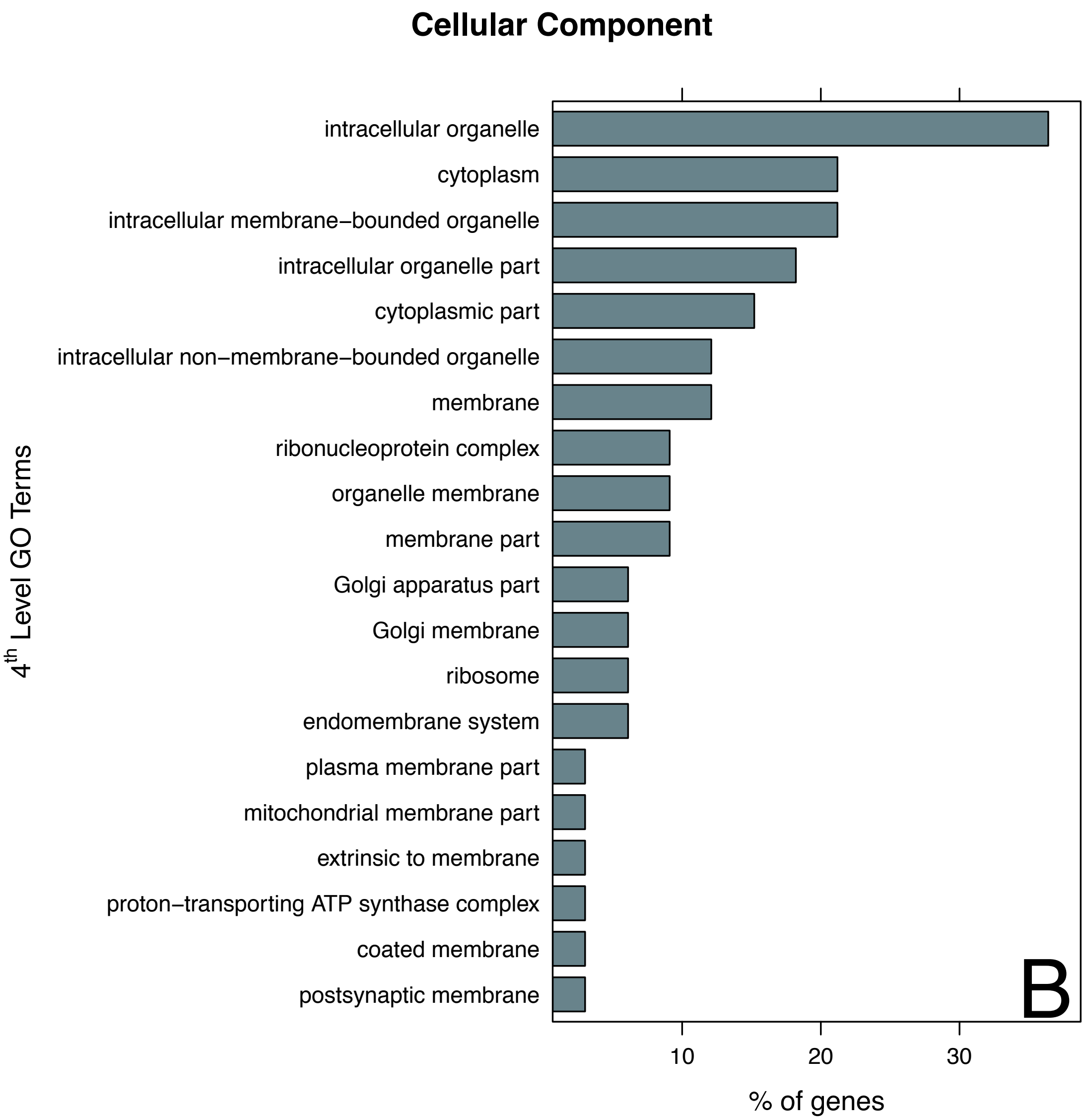
Cellular Component
intracellular organelle
cytoplasm
intracellular membrane−bounded organelle
intracellular organelle part
cytoplasmic part
intracellular non−membrane−bounded organelle
membrane
ribonucleoprotein complex
organelle membrane
membrane part
Golgi apparatus part
Golgi membrane
ribosome
endomembrane system
plasma membrane part
mitochondrial membrane part
extrinsic to membrane
proton−transporting ATP synthase complex
coated membrane
postsynaptic membrane
4th Level GO Terms
10
20
30
% of genes
B

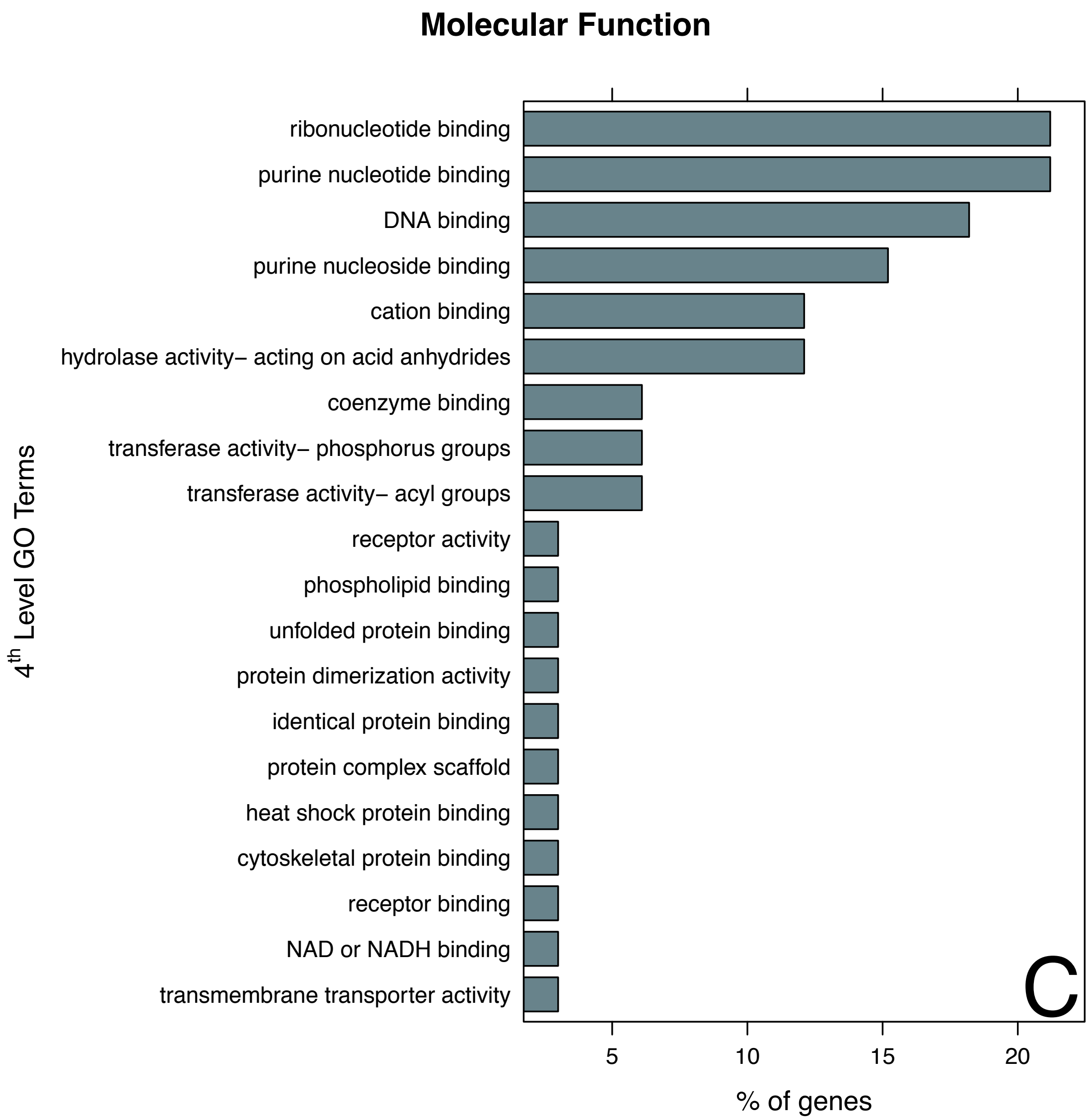

Figure S8 4th level GO term distribution for decreased rate genes. The top 20 most significant GO terms of each of the three ontologies, (a), (b), and (c).

Table S1 Accelerated genes and their gene products.
29 genes were shown to be evolving at an accelerated rate. Of these, four could be annotated. The *Enallagma* ORF, associated gene, its gene product, and GO ID's are shown.

ORF	Gene	Gene Product	Associated GO ID's
contig12757	Nol10	Nucleolar Protein 10	GO:0005730
contig13640	Art7	Protein arginine N-methyltransferase 7	GO:0005737 GO:0019918 GO:0035243
contig12629	Rrp45	mRNA processing	GO:0000178 GO:0005730 GO:0005829 GO:0051252 GO:0004532 GO:0017091 GO:0006364 GO:0005515 GO:0043928
contig03660	Uba3	Ubiquitin-like modifier activating enzyme 3	GO:0016881 GO:0008641 GO:0005524 GO:0045116

Tables S2-S5

Available for download at http://www.g3journal.org/lookup/suppl/doi:10.1534/g3.113.005637/-/DC1.

Table S2 Decreased rate annotations

Of the 169 genes evolving at altered rates, 140 of these were shown to be evolving at a decreased rate. Of these 169, 33 were mapped to unique GO IDs.

Table S3 Gene ID's

The data set of 634 orthologous protein coding gene groups. Only genes that were present in all 11 species were included in this data set.

Table S4 Annotated Orthologs

Of the 634 genes in the orthologous, protein-coding set, we were able to map 488 of these to at least one GO ID. These genes were mapped to 1669 GO IDs, in total, with 691 of these GO IDs being unique.

Table S5 Newbler assembler (v.2.3) parameters
