# Supplementary material for: Functional Annotation and Comparative Analysis of a Zygopteran Transcriptome
Source: G3 (Bethesda). 2013 Apr 1;3(4):763–70. doi: 10.1534/g3.113.005637 (PMC3618363; doi:10.1534/g3.113.005637)
Supplement: Supporting Information [file supp_g3.113.005637_FigureS1.pdf]

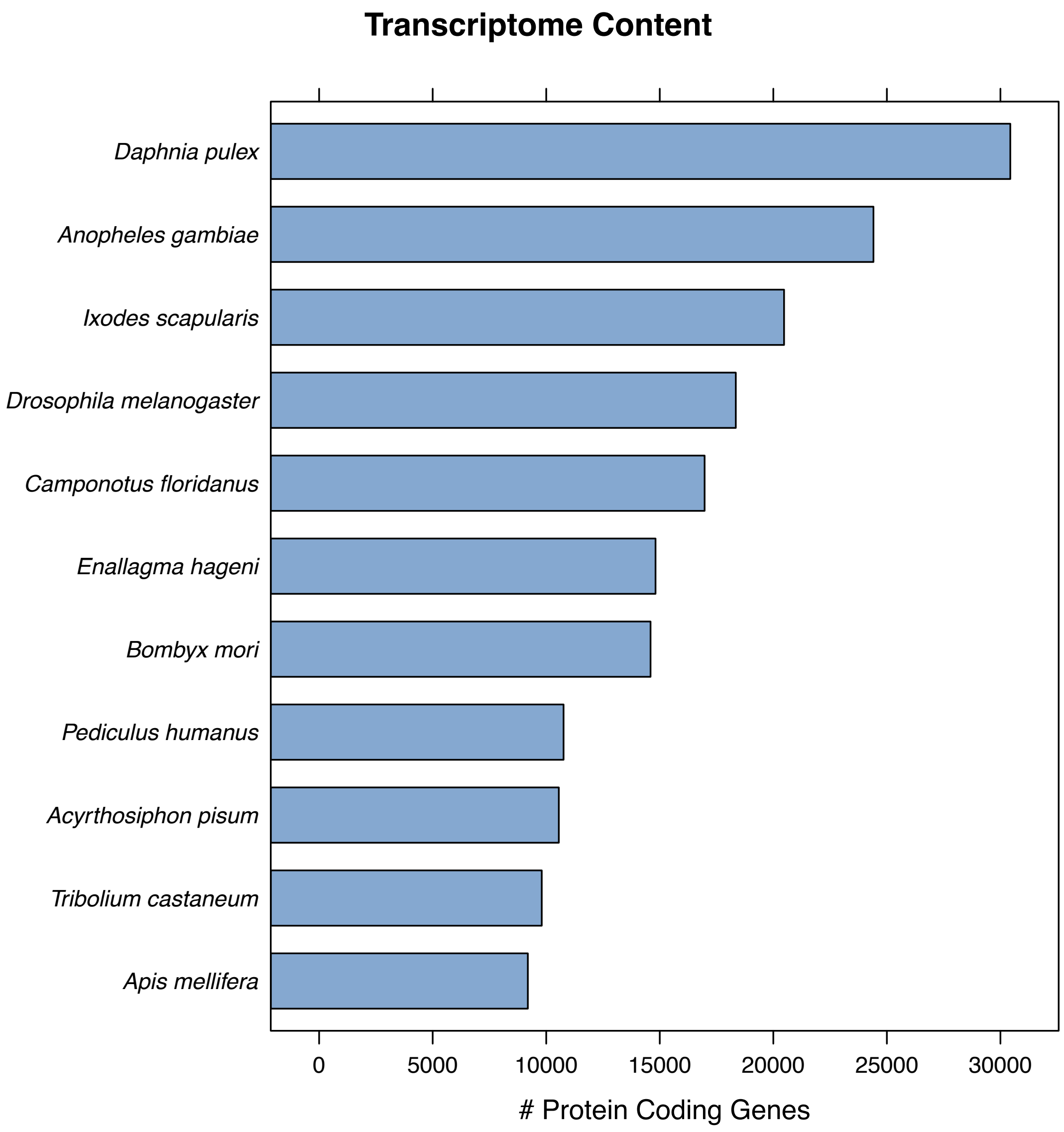

Figure S1 Transcriptome content. The number of protein coding genes of the 11 species used in our analysis is shown. The *Enallagma hageni* transcriptome possesses 14,813 protein coding genes.
