# Supplementary material for: Functional Annotation and Comparative Analysis of a Zygopteran Transcriptome
Source: G3 (Bethesda). 2013 Apr 1;3(4):763–70. doi: 10.1534/g3.113.005637 (PMC3618363; doi:10.1534/g3.113.005637)
Supplement: Supporting Information [file supp_g3.113.005637_FigureS2.pdf]

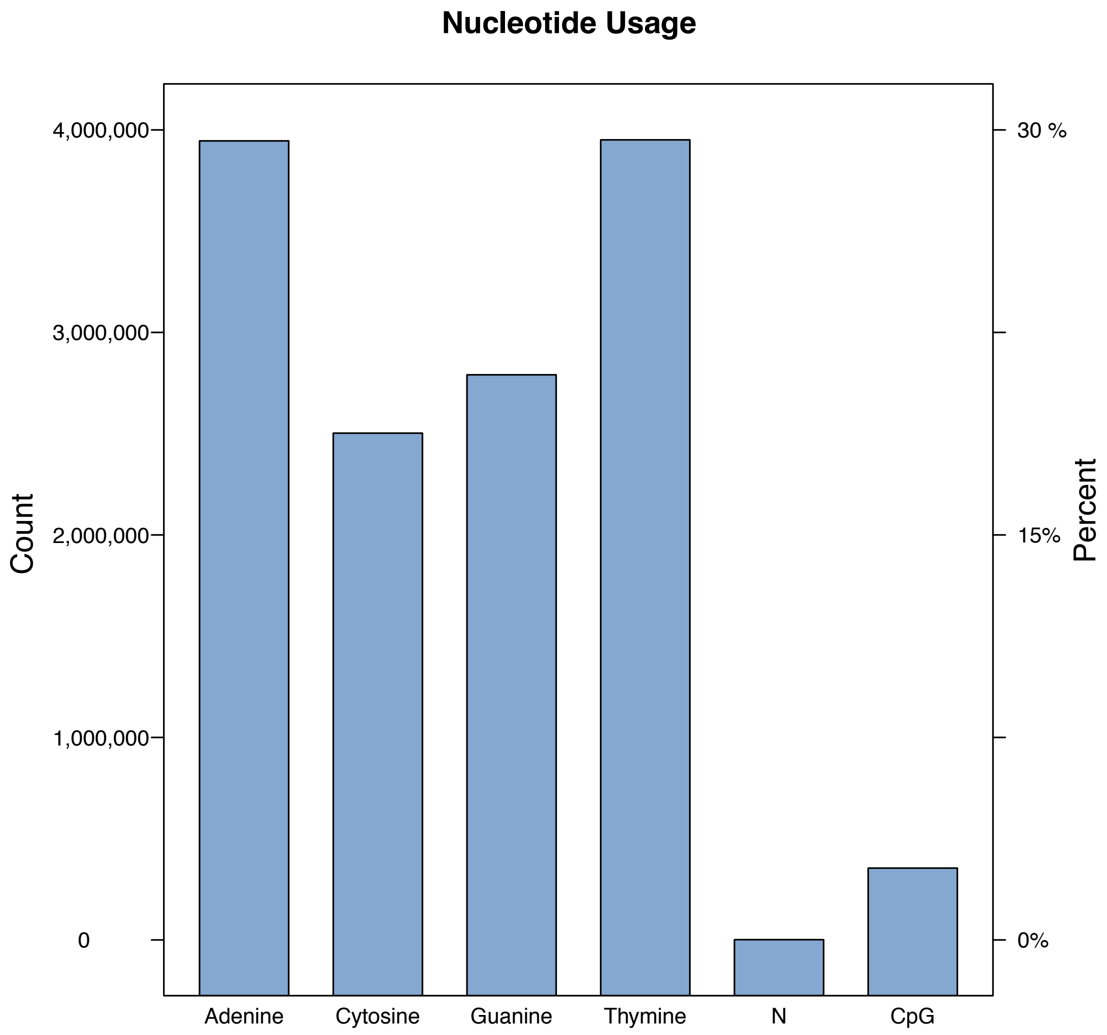


**Figure S2** Nucleotide profile of assembled *Enallagma* contigs. The assembled *E. hageni* transcriptome is comprised of 13,191,394 nucleotides. An AT bias is observed (59.86% AT, 40.13% GC, 0.01%N) and CpG sites occurred in 2.69% of the assembled transcriptome.
