# Supplementary material for: Functional Annotation and Comparative Analysis of a Zygopteran Transcriptome
Source: G3 (Bethesda). 2013 Apr 1;3(4):763–70. doi: 10.1534/g3.113.005637 (PMC3618363; doi:10.1534/g3.113.005637)
Supplement: Supporting Information [file supp_g3.113.005637_FigureS3.pdf]

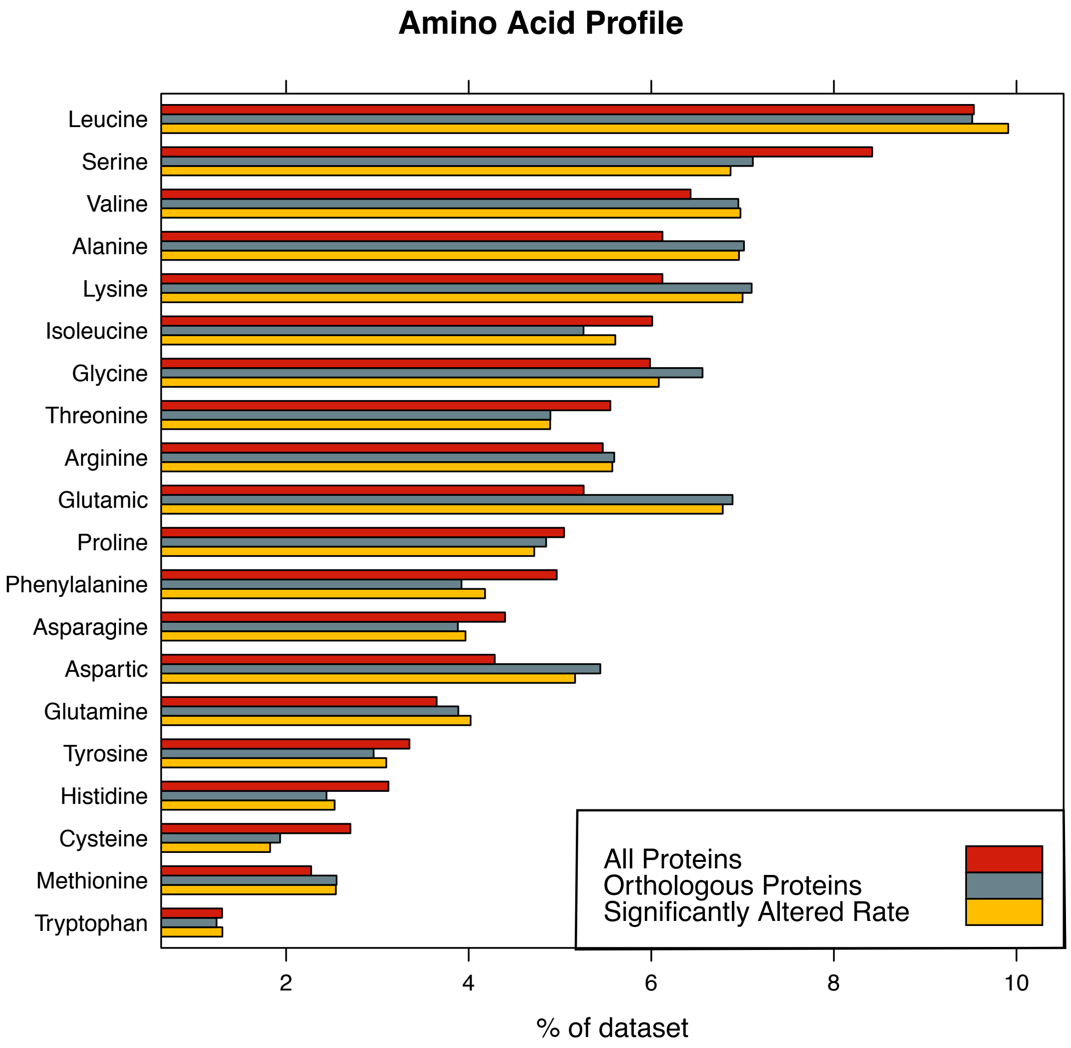

Figure S3 Amino acid profile. The amino acid profiles of three groups of translated *Enallagma* proteins are presented. The profile of all 1,621,208 amino acids comprising the 14,813 protein coding genes is shown in red. The 634 proteins orthologous across all 11 arthropod species in this study are indicated in grey and the 169 genes shown to have at an altered rate are shown in yellow.
