# Supplementary material for: Functional Annotation and Comparative Analysis of a Zygopteran Transcriptome
Source: G3 (Bethesda). 2013 Apr 1;3(4):763–70. doi: 10.1534/g3.113.005637 (PMC3618363; doi:10.1534/g3.113.005637)
Supplement: Supporting Information [file supp_g3.113.005637_FigureS4.pdf]

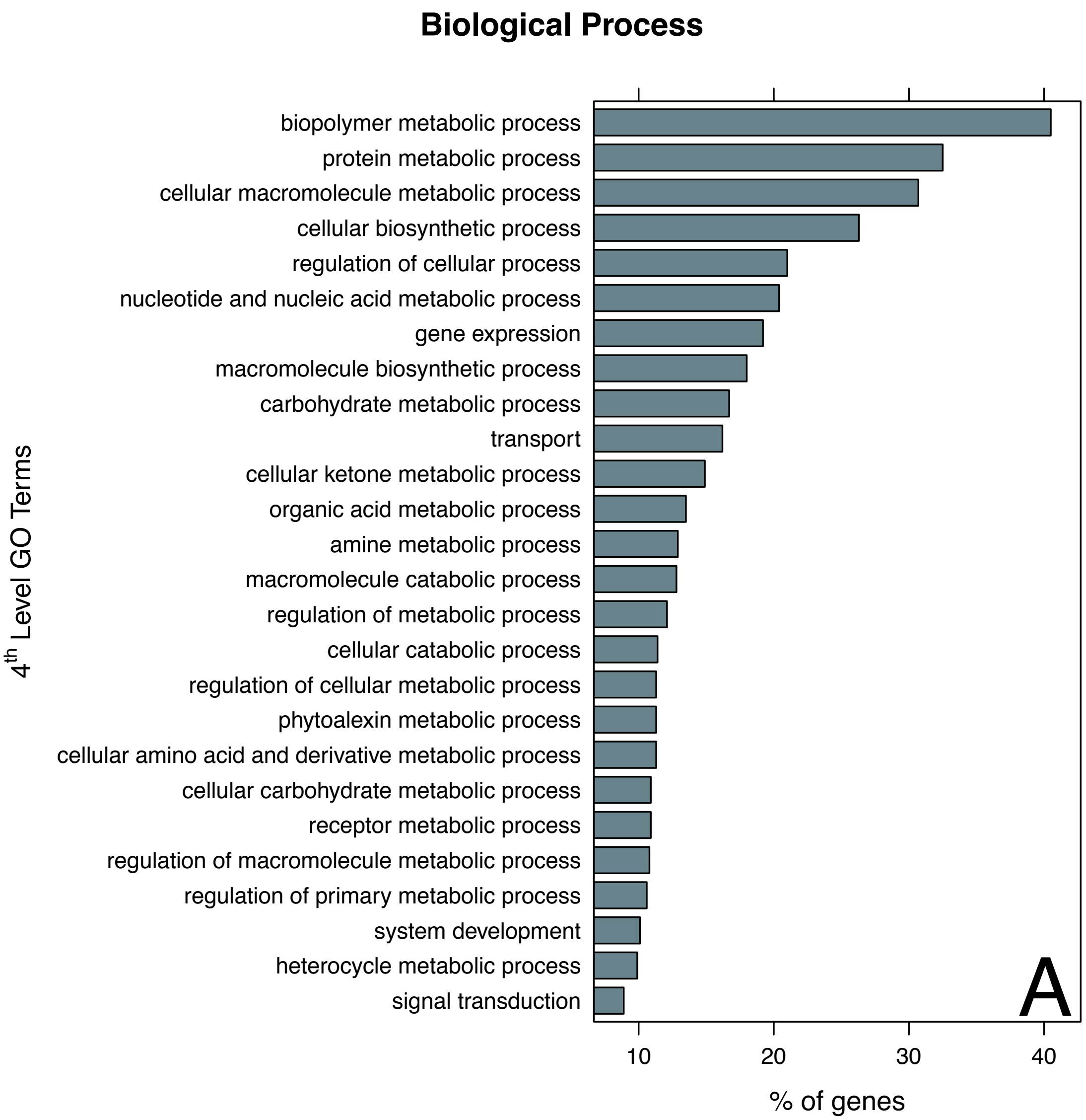
Biological Process
biopolymer metabolic process
protein metabolic process
cellular macromolecule metabolic process
cellular biosynthetic process
regulation of cellular process
nucleotide and nucleic acid metabolic process
gene expression
macromolecule biosynthetic process
carbohydrate metabolic process
transport
cellular ketone metabolic process
organic acid metabolic process
amine metabolic process
macromolecule catabolic process
regulation of metabolic process
cellular catabolic process
regulation of cellular metabolic process
phytoalexin metabolic process
cellular amino acid and derivative metabolic process
cellular carbohydrate metabolic process
receptor metabolic process
regulation of macromolecule metabolic process
regulation of primary metabolic process
system development
heterocycle metabolic process
signal transduction
4th Level GO Terms
10
20
30
40
% of genes
A

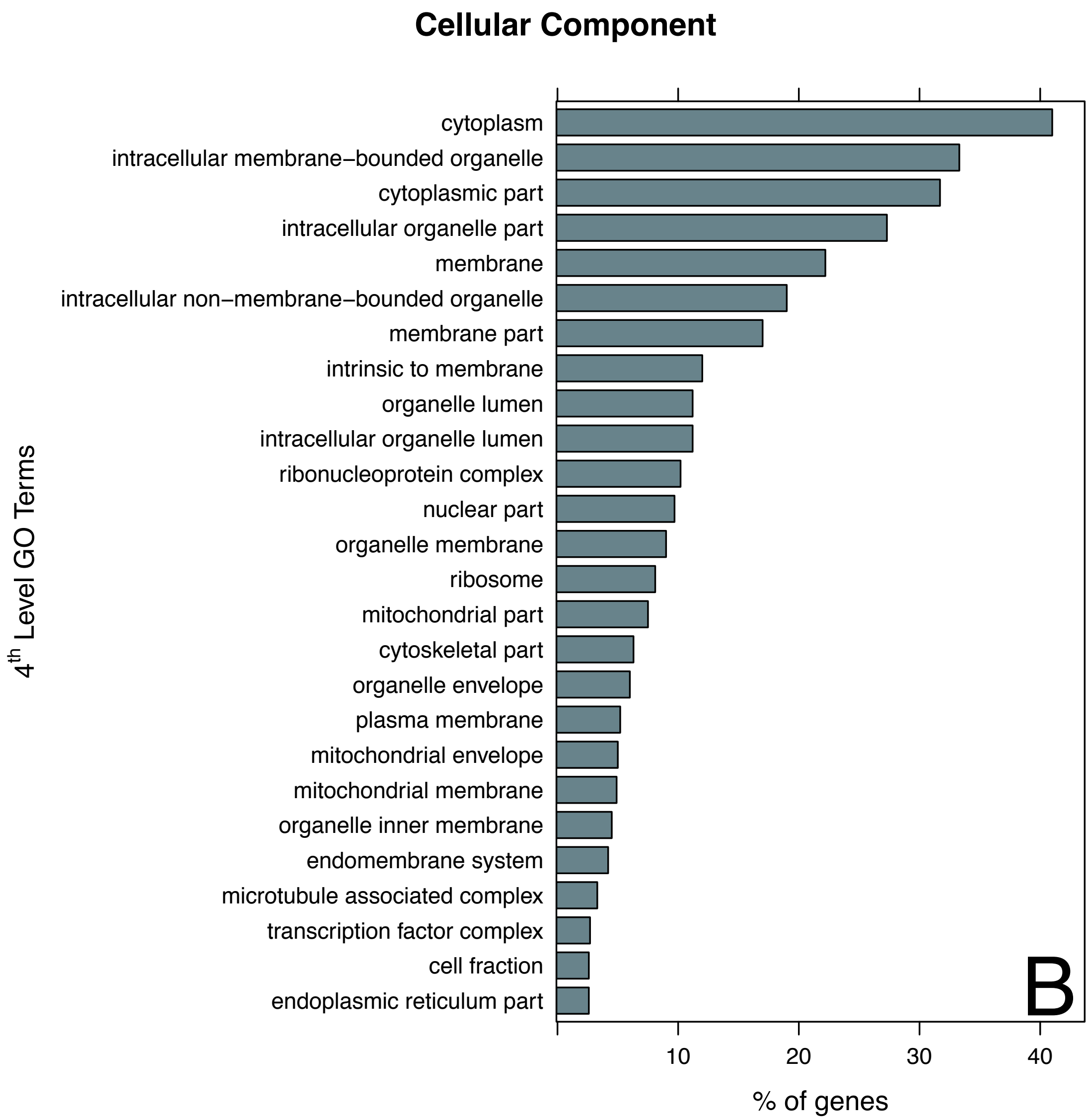

Cellular Component
cytoplasm
intracellular membrane−bounded organelle
cytoplasmic part
intracellular organelle part
membrane
intracellular non−membrane−bounded organelle
membrane part
intrinsic to membrane
organelle lumen
intracellular organelle lumen
ribonucleoprotein complex
nuclear part
organelle membrane
ribosome
mitochondrial part
cytoskeletal part
organelle envelope
plasma membrane
mitochondrial envelope
mitochondrial membrane
organelle inner membrane
endomembrane system
microtubule associated complex
transcription factor complex
cell fraction
endoplasmic reticulum part
4th Level GO Terms
10
20
30
40
% of genes
B

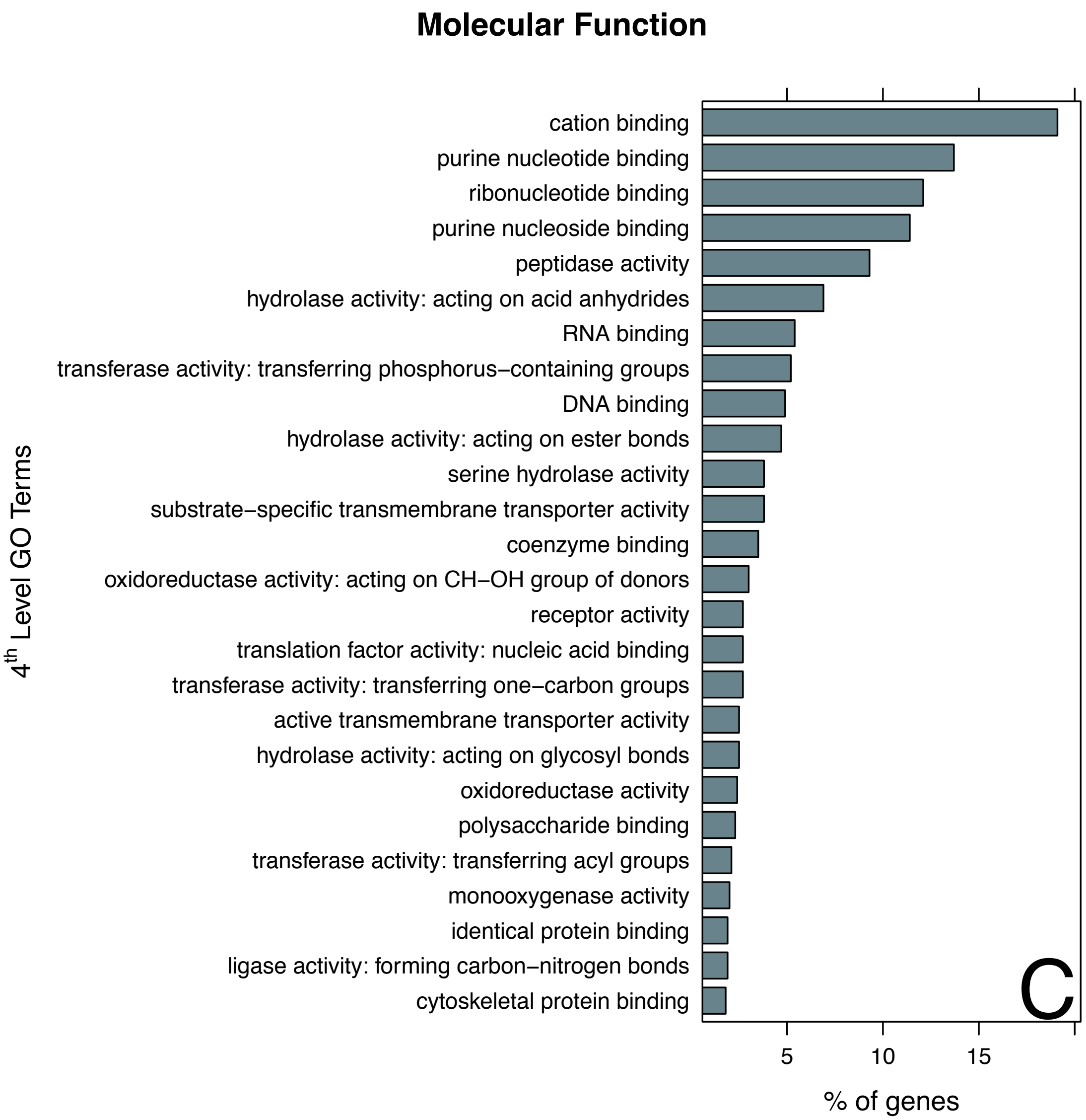

Figure S4 4th level GO term distributions for all annotated *Enallagma* genes. At the 4th level of the GO term hierarchy, we mapped the dataset of genes to 1463 GO terms across the 3 ontologies. Shown are the top 25 most significant results in each of the 1st level categories, A) biological processes, B) cellular components, and 3) molecular function.
