# Supplementary material for: Functional Annotation and Comparative Analysis of a Zygopteran Transcriptome
Source: G3 (Bethesda). 2013 Apr 1;3(4):763–70. doi: 10.1534/g3.113.005637 (PMC3618363; doi:10.1534/g3.113.005637)
Supplement: Supporting Information [file supp_g3.113.005637_FigureS5.pdf]

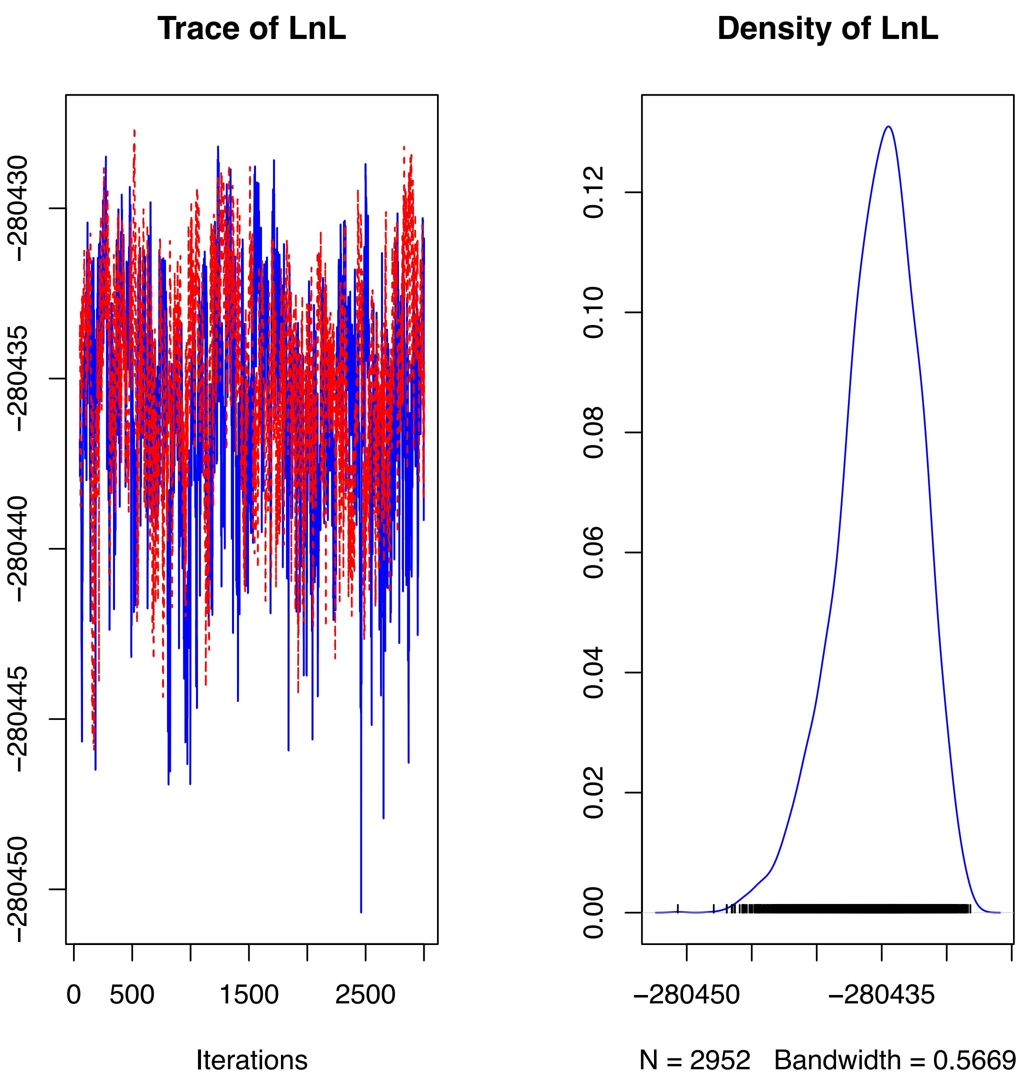


**Figure S5** Trace and density plots of the posterior probability of the phylogenetic analysis. After thinning the samples of the posterior probability, we obtained 2952 draws from the posterior. Shown in (A) is the negative log-likelihood trace plot. In (B) we plot the density of the thinned posterior.
