# Supplementary material for: Functional Annotation and Comparative Analysis of a Zygopteran Transcriptome
Source: G3 (Bethesda). 2013 Apr 1;3(4):763–70. doi: 10.1534/g3.113.005637 (PMC3618363; doi:10.1534/g3.113.005637)
Supplement: Supporting Information [file supp_g3.113.005637_FigureS6.pdf]

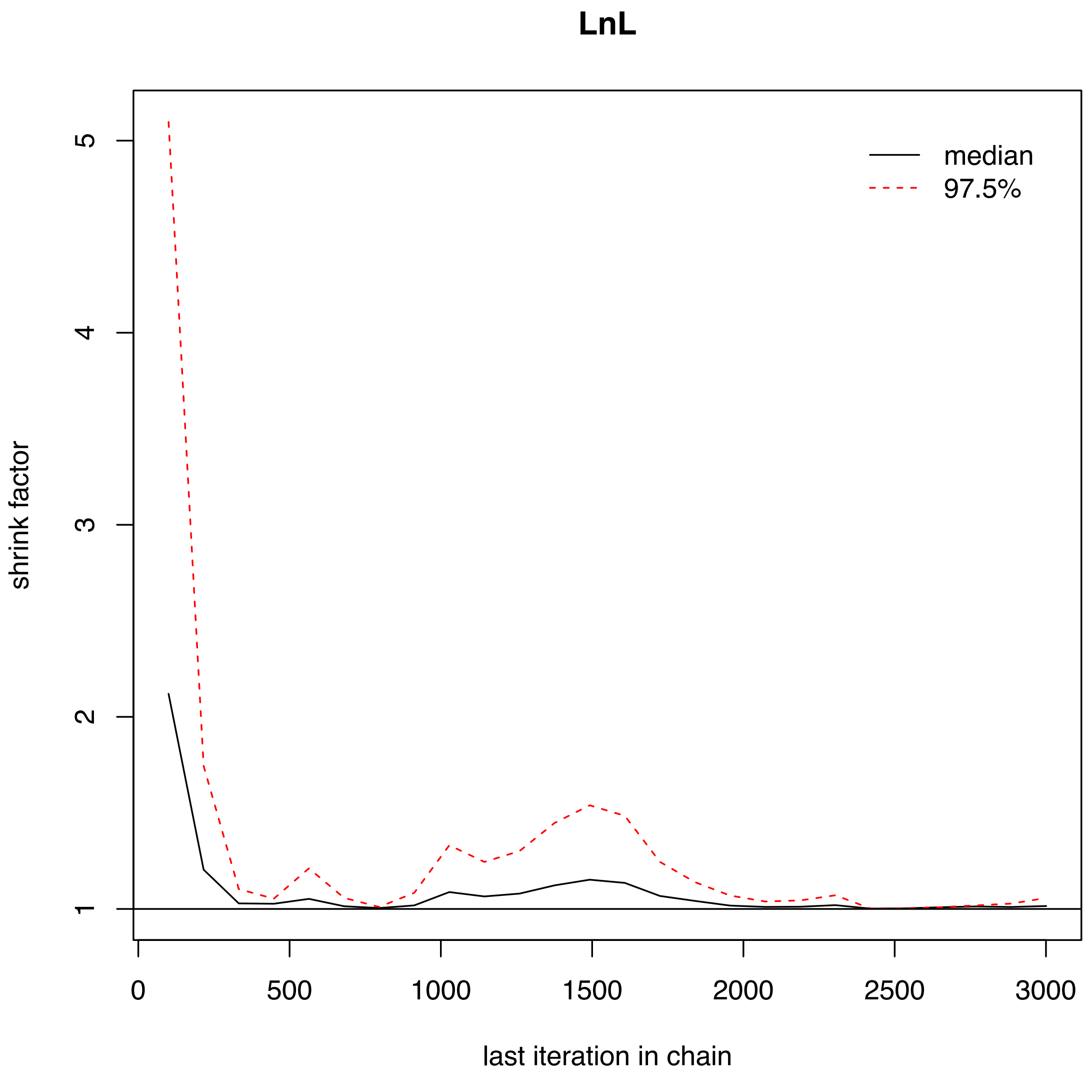

Figure S6 Gelman and Rubin convergence plot of the MCMC analysis. To test that our chains have converged to the stationary distribution, the thinned samples from both chains in the MCMC run are used to compute the Gelman and Rubin test for convergence. This test calculates the within-chain and between-chain variance and returns a potential scale reduction factor. If this factor is below ~1.25, it is another assurance that the stationary distribution has been reached.
