# Supplementary material for: Functional Annotation and Comparative Analysis of a Zygopteran Transcriptome
Source: G3 (Bethesda). 2013 Apr 1;3(4):763–70. doi: 10.1534/g3.113.005637 (PMC3618363; doi:10.1534/g3.113.005637)
Supplement: Supporting Information [file supp_g3.113.005637_FigureS7.pdf]

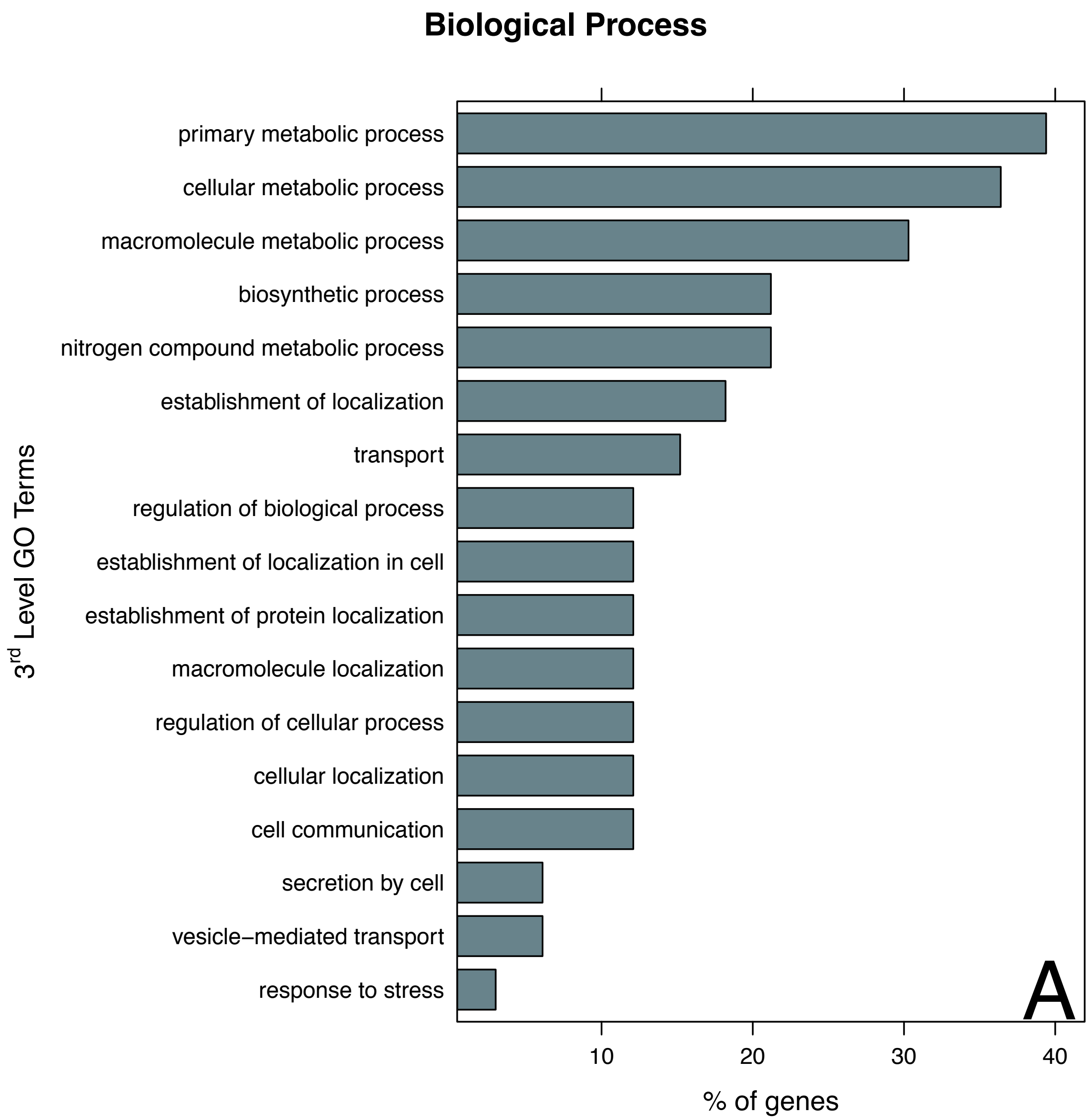

Biological Process
primary metabolic process
cellular metabolic process
macromolecule metabolic process
biosynthetic process
nitrogen compound metabolic process
establishment of localization
transport
regulation of biological process
establishment of localization in cell
establishment of protein localization
macromolecule localization
regulation of cellular process
cellular localization
cell communication
secretion by cell
vesicle–mediated transport
response to stress
3rd Level GO Terms
A
10
20
30
40
% of genes

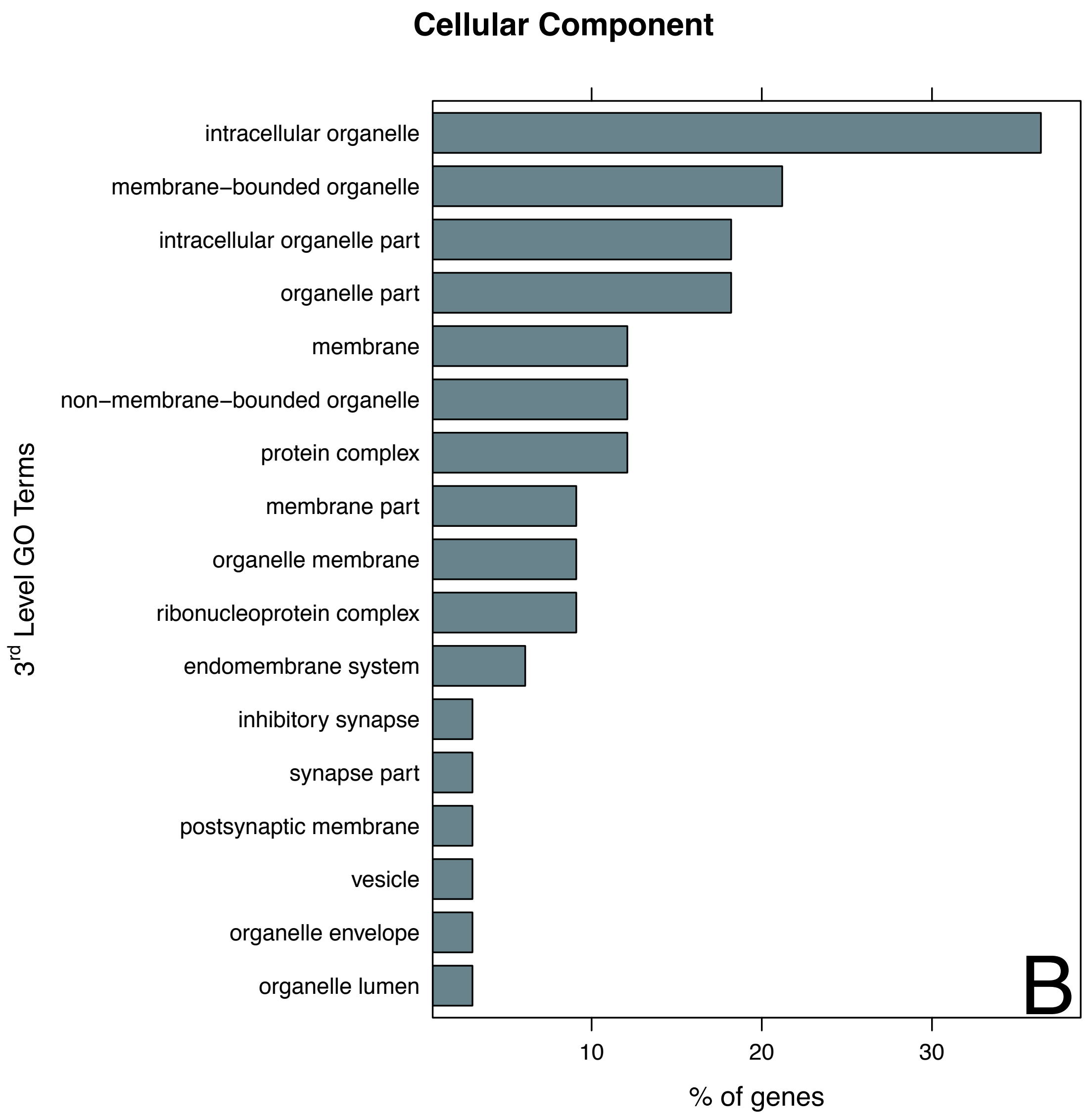
Cellular Component
intracellular organelle
membrane−bounded organelle
intracellular organelle part
organelle part
membrane
non−membrane−bounded organelle
protein complex
membrane part
organelle membrane
ribonucleoprotein complex
endomembrane system
inhibitory synapse
synapse part
postsynaptic membrane
vesicle
organelle envelope
organelle lumen
3rd Level GO Terms
10
20
30
% of genes
B

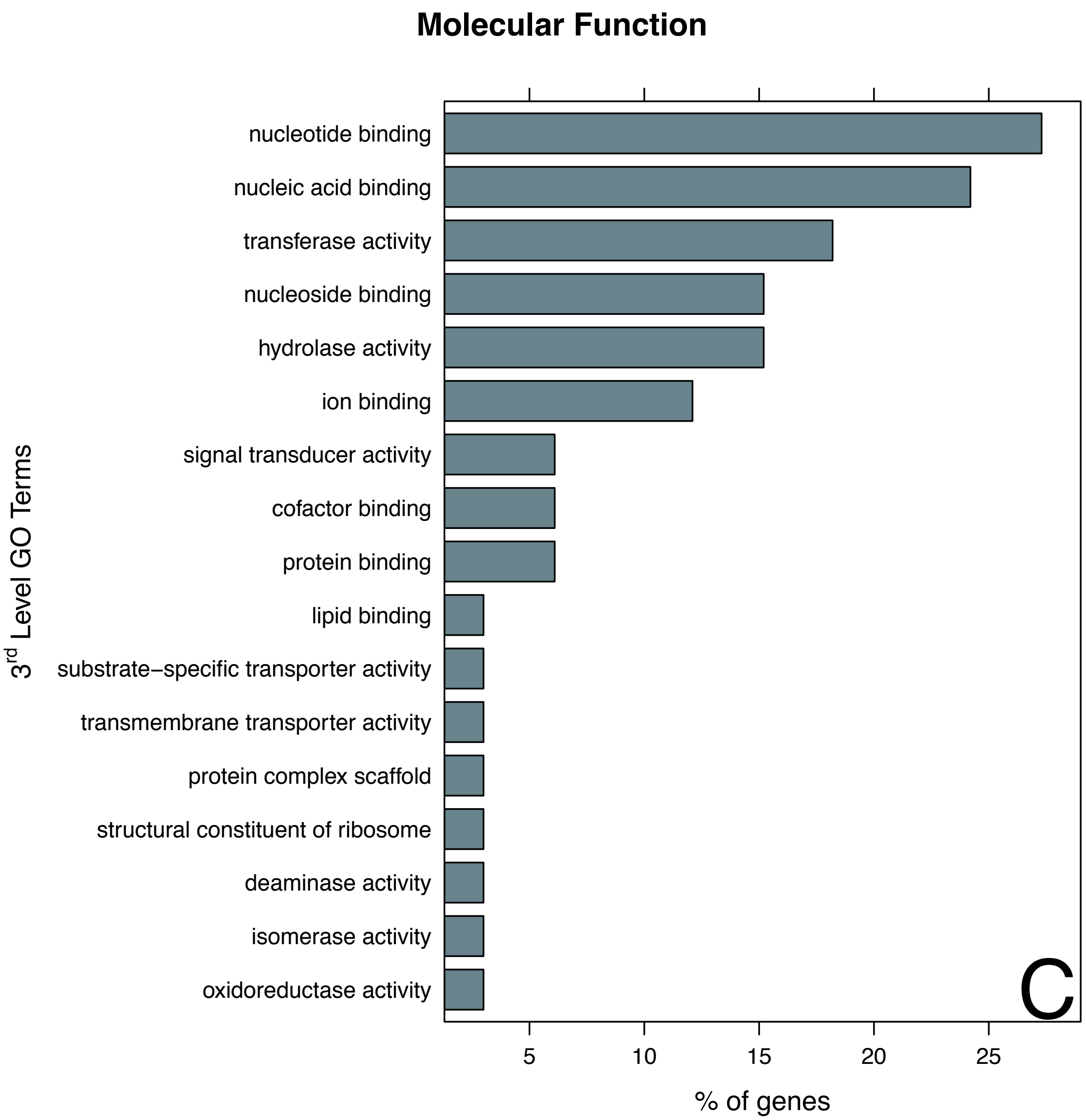

Figure S7 3rd level GO term distribution for decreased rate genes. Of the 140 *Enallagma hageni* genes which were shown to be evolving at either a diminished rate, per the branch length tests, we were able to map 33 of these genes to 105 GO terms. Shown here are the top 17 most significant of these terms across the three orthologies, (a),(b), and (c).
