# Supplementary material for: Functional Annotation and Comparative Analysis of a Zygopteran Transcriptome
Source: G3 (Bethesda). 2013 Apr 1;3(4):763–70. doi: 10.1534/g3.113.005637 (PMC3618363; doi:10.1534/g3.113.005637)
Supplement: Supporting Information [file supp_g3.113.005637_FigureS8.pdf]

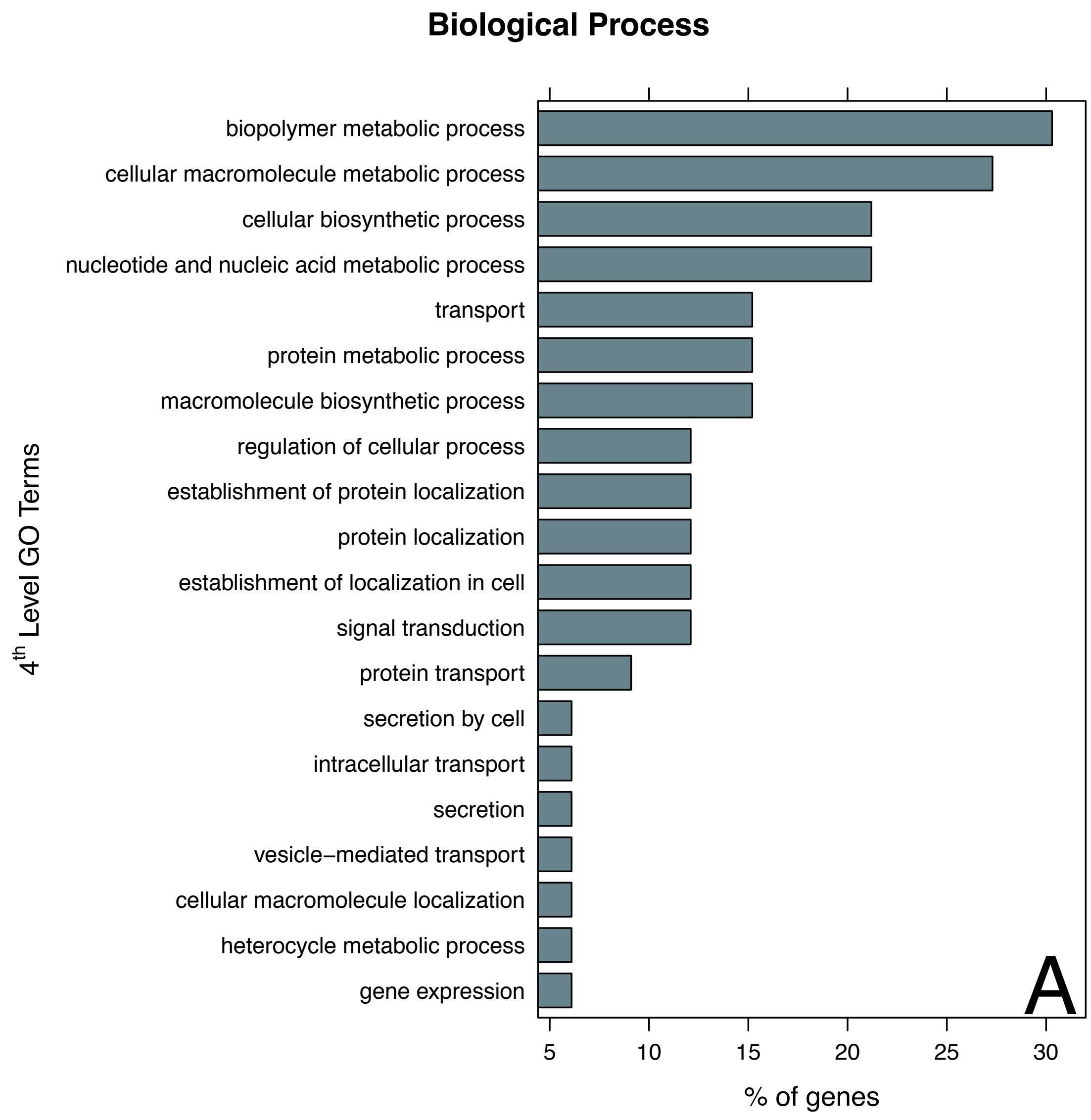
Biological Process
biopolymer metabolic process
cellular macromolecule metabolic process
cellular biosynthetic process
nucleotide and nucleic acid metabolic process
transport
protein metabolic process
macromolecule biosynthetic process
regulation of cellular process
establishment of protein localization
protein localization
establishment of localization in cell
signal transduction
protein transport
secretion by cell
intracellular transport
secretion
vesicle−mediated transport
cellular macromolecule localization
heterocycle metabolic process
gene expression
4th Level GO Terms
5
10
15
20
25
30
% of genes
A

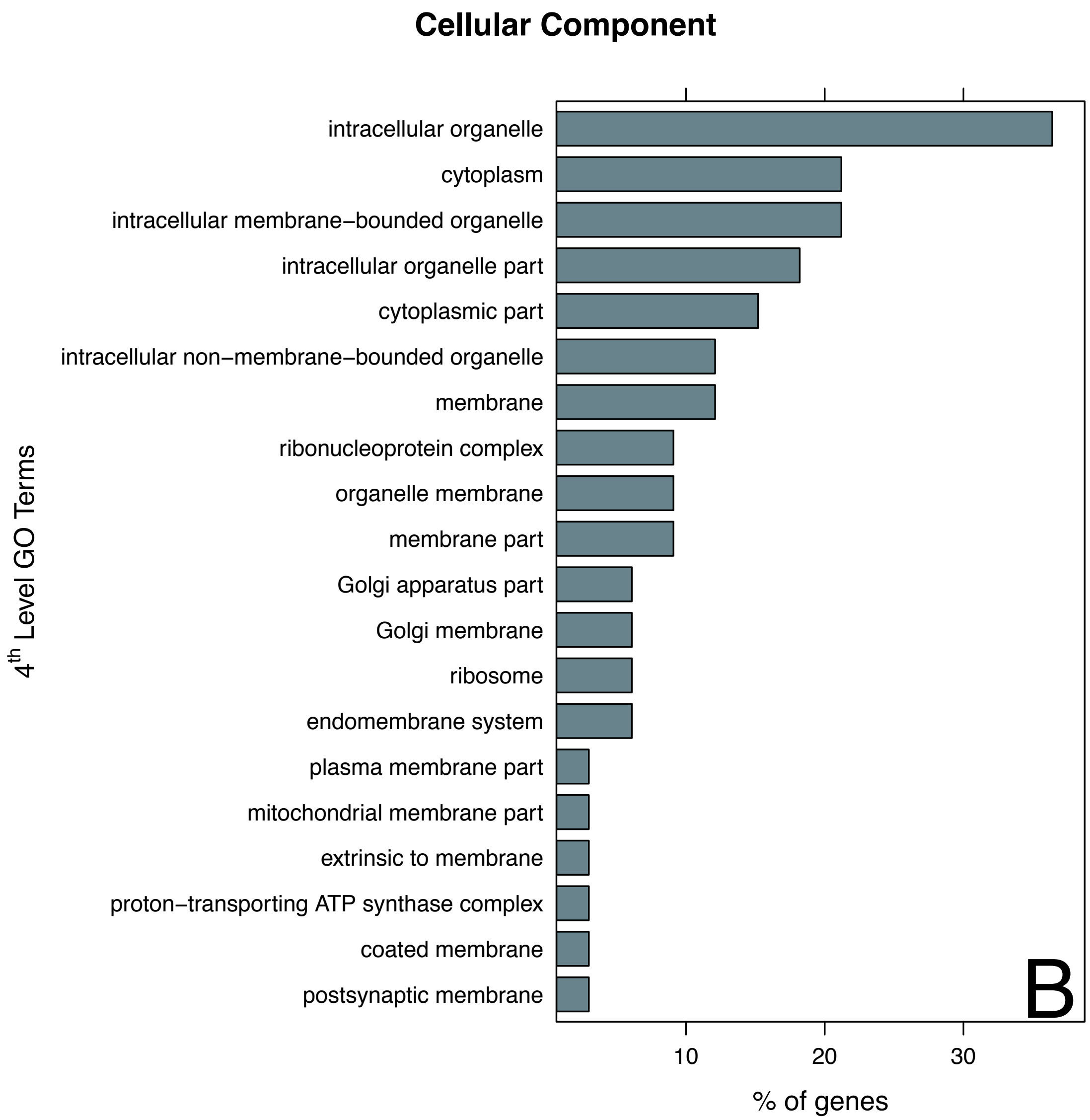

Cellular Component
intracellular organelle
cytoplasm
intracellular membrane−bounded organelle
intracellular organelle part
cytoplasmic part
intracellular non−membrane−bounded organelle
membrane
ribonucleoprotein complex
organelle membrane
membrane part
Golgi apparatus part
Golgi membrane
ribosome
endomembrane system
plasma membrane part
mitochondrial membrane part
extrinsic to membrane
proton−transporting ATP synthase complex
coated membrane
postsynaptic membrane
B
4th Level GO Terms
10
20
30
% of genes

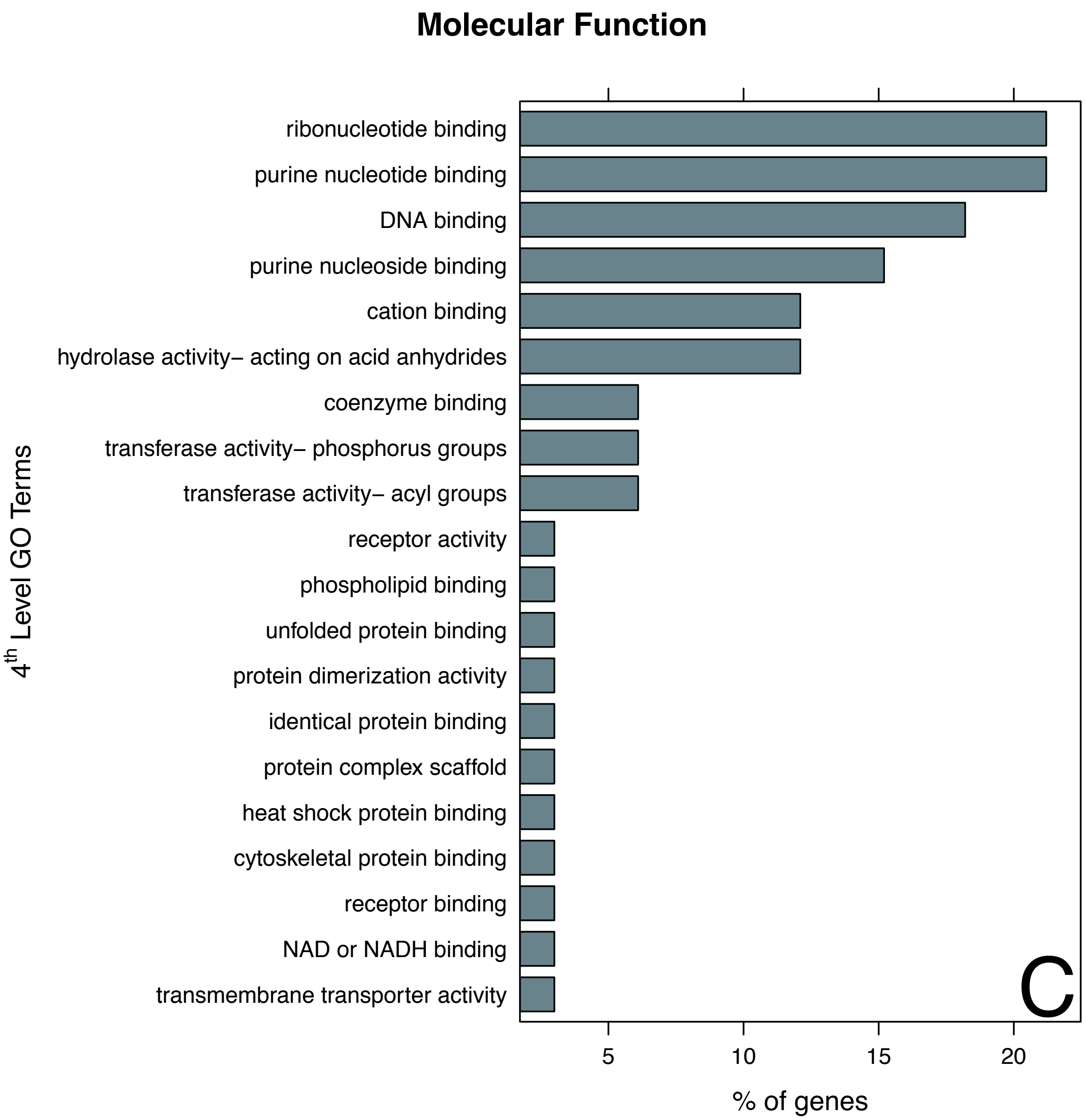

Figure S8 4th level GO term distribution for decreased rate genes. The top 20 most significant GO terms of each of the three ontologies, (a), (b), and (c).
