# Supplementary material for: Functional Annotation and Comparative Analysis of a Zygopteran Transcriptome
Source: G3 (Bethesda). 2013 Apr 1;3(4):763–70. doi: 10.1534/g3.113.005637 (PMC3618363; doi:10.1534/g3.113.005637)
Supplement: Supporting Information [file supp_g3.113.005637_TableS1.pdf]

Table S1 Accelerated genes and their gene products.

29 genes were shown to be evolving at an accelerated rate. Of these, four could be annotated. The *Enallagma* ORF, associated gene, its gene product, and GO ID's are shown.

ORF	Gene	Gene Product	Associated GO ID's
contig12757	Nol10	Nucleolar Protein 10	GO:0005730
contig13640	Art7	Protein arginine N-methyltransferase 7	GO:0005737 GO:0019918 GO:0035243
contig12629	Rrp45	mRNA processing	GO:0000178 GO:0005730 GO:0005829 GO:0051252 GO:0004532 GO:0017091 GO:0006364 GO:0005515 GO:0043928
contig03660	Uba3	Ubiquitin-like modifier activating enzyme 3	GO:0016881 GO:0008641 GO:0005524 GO:0045116
